# Enabling mRNA Therapeutics: Current Landscape and Challenges in Manufacturing

**DOI:** 10.3390/biom13101497

**Published:** 2023-10-09

**Authors:** Maryam Youssef, Cynthia Hitti, Julia Puppin Chaves Fulber, Amine A. Kamen

**Affiliations:** Department of Bioengineering, McGill University, Montreal, QC H3A 0G4, Canada; maryam.youssef@mail.mcgill.ca (M.Y.); cynthia.hitti@mail.mcgill.ca (C.H.); julia.puppinchavesfulber@mail.mcgill.ca (J.P.C.F.)

**Keywords:** mRNA therapeutics, mRNA manufacturing, in vitro transcription, lipid nanoparticles

## Abstract

Recent advances and discoveries in the structure and role of mRNA as well as novel lipid-based delivery modalities have enabled the advancement of mRNA therapeutics into the clinical trial space. The manufacturing of these products is relatively simple and eliminates many of the challenges associated with cell culture production of viral delivery systems for gene and cell therapy applications, allowing rapid production of mRNA for personalized treatments, cancer therapies, protein replacement and gene editing. The success of mRNA vaccines during the COVID-19 pandemic highlighted the immense potential of this technology as a vaccination platform, but there are still particular challenges to establish mRNA as a widespread therapeutic tool. Immunostimulatory byproducts can pose a barrier for chronic treatments and different production scales may need to be considered for these applications. Moreover, long-term storage of mRNA products is notoriously difficult. This review provides a detailed overview of the manufacturing steps for mRNA therapeutics, including sequence design, DNA template preparation, mRNA production and formulation, while identifying the challenges remaining in the dose requirements, long-term storage and immunotolerance of the product.

## 1. Introduction

From the first demonstration of messenger RNA (mRNA) delivery into in vivo models [1] to the development of lipid nanoparticles using ionizable lipids as a delivery system for the first approved siRNA therapeutic [2], decades of fundamental research converged to enable the creation of feasible RNA vaccines and therapeutics. Given the global success demonstrated during the COVID-19 pandemic, the mRNA technology platform is currently in the spotlight and shows considerable potential not only for vaccines, but also for treatment of diseases [3,4,5,6,7]. This promising technology was shown to have incredibly fast development, manufacturing, and roll-out times [8], with the potential of revolutionizing the therapeutic field as a drug with a wide range of applications [9].

mRNA technology relies on the transfer of an mRNA either encoding an antigen or a therapeutic protein of interest into the cytoplasm [3]. This genetic message, once translated, functions to stimulate an immune response or alter a disease state. In contrast to DNA therapeutics, mRNA does not require nuclear entry as expression occurs directly in the cytoplasm. In addition, this eliminates the risk of insertional oncogenesis associated with viral vector and plasmid DNA techniques. Their ability to be manufactured through cell-free systems eliminates the time constraints and possible contaminants associated with traditional viral vector-based gene and cell therapies [10,11]. Therapeutic use of adeno-associated viruses, for example, require high doses that are difficult to accommodate with the current productivity and scalability issues [12], while enzymatic mRNA production is linearly scalable [13], thus achieving the required production more easily. Moreover, a new mRNA product implies a new sequence, but the physicochemical characteristics of an mRNA remain the same, meaning that the manufacturing process can be replicated with minimal changes for a powerful plug-and-play platform [14]. The technology’s versatility allows it a broad potential of therapeutic applications including but not limited to cell reprogramming, gene editing, protein replacement therapy and cancer immunotherapy [3].

**Figure 1:**
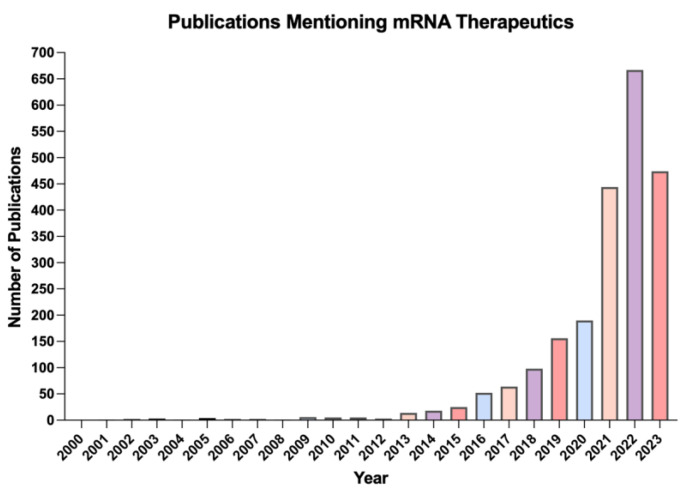
Yearly number of publications mentioning mRNA therapeutics over the past two decades, as of August 2023.

Historically, the progression of RNA-based gene and cell therapies has been limited by challenges related to the instability of RNA, the immunogenic response to RNA molecules as well as the delivery of the RNA across the cell membrane. However, several improvements have been made to increase the stability and to reduce the degradation of RNA molecules including base modifications and the emergence of the lipid nanoparticle (LNP) as an advanced tool for the delivery of RNA-based therapeutics [15]. A range of these drugs have reached the pre-clinical and clinical spaces, with the American Society for Gene and Cell Therapy reporting 897 RNA therapeutics in the clinical pipeline as of April 2023 [16]. In addition, the number of publications which mention mRNA therapeutics has been steadily increasing over the past decade, indicating the growth of the field, as shown in Figure 1.

Despite the technology’s potential, mRNA therapeutics have not taken the market by storm as quickly as the vaccines due mainly to different regulatory processes during global health emergencies. Moving forward, mRNA therapeutics will face challenges regarding different manufacturing and regulatory considerations compared to vaccines. The diversity of applications, classifications, and manufacturing protocols when it comes to mRNA therapeutics make streamlining of regulatory approval more difficult [17]. Moreover, immunostimulatory by-products such as double-stranded RNA must be strictly controlled during manufacturing to ensure immunotolerance of the drug for long-term mRNA treatments [18]. Currently, these therapeutics lack a standardized production pipeline across the published literature and within patents, and manufacturing scalability is limited by high costs associated with current good manufacturing practice (cGMP)-grade reagents required for in vitro transcription (IVT) [19]. Long-term treatments with repeated dosing requires affordable drugs of consistent yield and quality.

As such, this review addresses the challenges to enable widespread use of mRNA therapeutics from a manufacturing perspective, focusing on particularities of therapeutics in contrast to what has been established with production and approval of mRNA vaccines. First, we explore the current landscape of applications in the clinical trial space. Next, we discuss the entire manufacturing pipeline, including sequence design and optimization; production and purification of template DNA; production and purification of mRNA; microfluidic techniques for encapsulation in LNPs; and formulation for product stability. Finally, we identify future perspectives in manufacturing and process automation, as well as novel strategies for delivery and targeting.

## 2. Current Therapeutic Applications of mRNA

In the context of gene and cell therapy, mRNA may be applied to replace or supplement disease genes and proteins. Specifically, mRNA as a tool for the restoration of a gene or protein in monogenic disorders is of interest within the field [20]. For example, a study conducted by Ramaswamy et al. demonstrated the delivery of factor IX encoding mRNA for the treatment of hemophilia B in a mouse model [21]. Similarly, An et al. demonstrated mRNA therapy in mice for the treatment of methylmalonic acidemia in mice using mRNA encoding human methylmalonyl-CoA mutase [22]. There are also several studies which discuss the use of mRNA therapy for cardiac related diseases [23]. For example, Zangi et al. explored the use of mRNA encoding the vascular endothelial growth factor A gene for cardiac tissue repair [24].

Furthermore, there is therapeutic value in the use of mRNA in cancer treatments. In these cases, the mRNA can be used to deliver suicide genes or tumor-associated antigens (TAAs). A 2019 study examined the use of mRNA encoding a suicide gene for colon cancer therapy and was found to be successful at shrinking tumor size in mice models [25]. Several mRNA treatments for rare genetic diseases have also made it into the clinical pipeline in recent years, with companies such as Moderna and Translate Bio occupying the space [26].

Future prospects in the development of personalized gene therapies are promising due to the rapid manufacturing of these molecules. Personalized medicine strategies consider each patients’ genotypic characteristics, which will allow the production of therapies specifically for cancer patients, rare metabolic disease patients and a range of other pathologies [27,28]. From a manufacturing perspective, these personalized medicines may require smaller infrastructure than that established throughout the COVID-19 pandemic due to the scale of production for individual patients rather than global populations. Hospital-based RNA therapeutic programs, to expedite the process of genetic testing to the production of small cGMP grade material, may accommodate the implementation of these personalized therapeutics [29].

Cell therapies, traditionally performed using viral vectors, have also been an active area of development using RNA therapeutics. Specifically, chimeric antigen receptor (CAR) T-cell therapy has been of interest [30]. This process involves the delivery of the CAR to autologous cells using mRNA and re-infusing these cells to the patient after their modification. Several clinical trials have been performed using this method, including ECI-006, MCY-M11 and Descartes-08, which target melanoma, mesothelin-expressing solid tumors and multiple myeloma, respectively [31].

Similarly, the use of in vitro transcribed mRNA for gene editing techniques is growing both in the literature reports and in the clinical applications. This technique involves mRNA which encodes genome editing nucleases, including zinc finger nucleases, CRISPR-associated nucleases and transcription activator-like effector nucleases, and a guide RNA to repair DNA mutations [32]. The mRNA expresses the encoded nuclease in the cytoplasm, which then enters the nucleus alongside the guide RNA. As such, this technique avoids the challenge associated with DNA-mediated approaches of delivering the encoded nuclease genes to the nucleus before expression and translation in the cytoplasm [32]. Several clinical trials have applied this technique, including a trial sponsored by the University of Pennsylvania for the treatment of HIV-1 infected patients [33]. This technology has been successful in primates, as demonstrated by Munusuru et al. in a recent study [34], and is currently being evaluated in clinical trials conducted by Intellia therapeutics [35,36].

### Ongoing Clinical Trials

**Figure 2:**
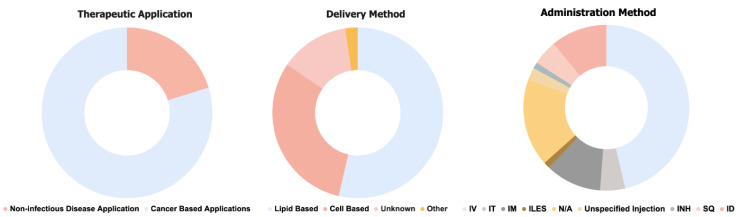
Distribution of ongoing clinical trials by application, delivery method and administration method. IV: intravenous, IT: intratumoral, IM: intramuscular, ILES: intralesional, SQ: subcutaneous, INH: inhalation, ID: intradermal, N/A: Not Available.

Currently, there are 84 ongoing (not yet recruiting, recruiting, and active) trials evaluating mRNA therapeutics, with the majority targeting cancer (Figure 2). The majority of ongoing clinical trials involve mRNA therapeutics employing the use of lipid nanoparticles or liposomes. Among these lipid-based mRNA therapeutics, a wide variety of treatments are currently being explored, including protein replacement therapy [37,38,39], cancer immunotherapy, personalized cancer vaccines [40,41,42,43], mRNA-encoded monoclonal antibodies [44,45], and gene editing [35,36] (Table 1). This demonstrates the lipid nanoparticle (LNP) technology’s versatility.

Despite the large range of applications, the majority of the lipid-based mRNA therapeutics in ongoing clinical trials are administered intravenously. For immunotherapeutic applications, intravenous administration allows for high antigen production levels as opposed to other administration methods [46]. The advantage of systemic delivery is, however, accompanied by the requirement of manufacturing larger dosages as opposed to tissue-specific delivery. Intravenous administration requires larger volumes of the drug, and typically leads to the accumulation of the drug in the liver [47], which may limit their internalization in the target tissue. For example, where many therapeutics delivered by IV have been evaluated at mRNA doses in the milligram range [48,49,50,51], an intratumorally delivered drug, MEDI1191, was evaluated at mRNA dosages of 0.1–12 μg [52]. Another targeted administration method, inhalation, has also presented potential for both therapeutic and vaccine applications in recent years. Recent advancements in the nebulization and formulation of lipid nanoparticle products for inhalation demonstrate the possibility of an increase in the number of these products [53,54]. Currently, however, ARCT-032 and VX-522, protein replacement therapies for cystic fibrosis, are the only inhalable mRNA therapeutics in ongoing trials [55,56].

Additionally, the need for repeated dosing is clearly illustrated in these current ongoing clinical trials, emphasizing the need for high-yield production processes for mRNA drug products. However, the challenge of chronic dosing of mRNA therapeutics is balanced by its safety profile compared to viral vector therapeutics and its ability to produce high levels of protein intracellularly compared to protein therapeutics [4].

Beyond lipid-based delivery systems, several ongoing clinical trials are currently evaluating the use of dendritic cells pulsed with mRNA for cancer immunotherapy [57,58,59,60,61,62,63,64,65,66,67], mRNA-based T-cell therapies [68,69,70,71,72,73,74], and other carriers including exosomes and VLPs [75,76]. However, this review focuses on the manufacturing of lipid-based mRNA therapeutics due to their ubiquity in the clinical space.

## 3. Manufacturing Process

mRNA is a negatively charged, single-stranded molecule involved in protein synthesis that typically ranges between 1 and 15 kilobases in length (kb) [77,78]. It consists of a single-stranded open reading frame flanked by untranslated regions (5′-UTR and 3′-UTR), as well as 5′ cap and 3′ polyadenylation (poly(A)) tail sequences [27]. Each of these segments plays an essential role in the function and stability of the mRNA and must be incorporated throughout production. It is also possible to design a cap-independent RNA treatment by using circular RNA and internal ribosome entry sites [79,80,81,82], which implies a separate category of design and manufacturing considerations outside the scope of this review.

**Figure 3:**
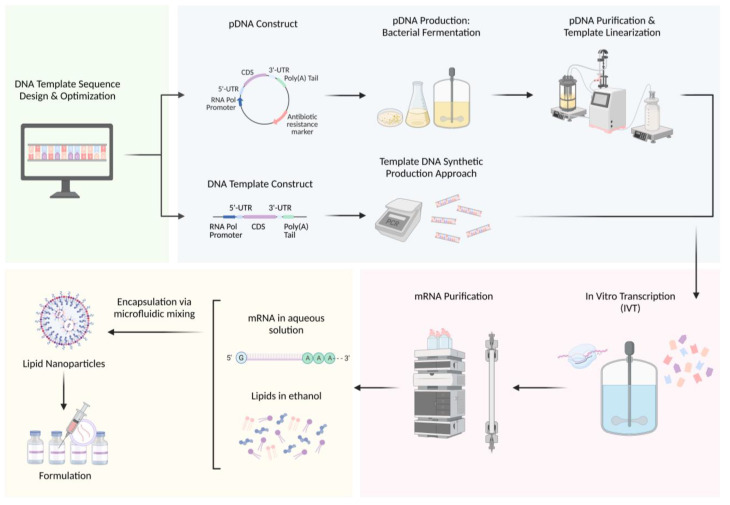
Manufacturing process for mRNA therapeutics: (1) DNA template sequence design and optimization, (2) DNA template production and purification using either bacterial fermentation or synthetic approaches, (3) mRNA synthesis via IVT, (4) mRNA purification, and (5) encapsulation for delivery and storage.

The production process of a new mRNA therapeutic can be divided into the following main steps: (1) DNA template sequence design, (2) DNA template production and purification, (3) IVT, (4) mRNA purification, and (5) encapsulation and formulation for delivery and storage, as illustrated in Figure 3.

### 3.1. Upstream Process: DNA Template Sequence Design

The production process of mRNA therapeutics begins with the design of a DNA template for subsequent IVT. Transcription templates for mRNA synthesis can be in the form of plasmid DNA (pDNA), PCR products or synthetic double-stranded oligonucleotides [83,84]. Typically, the DNA template should include the following elements [85]: promoter sequence, gene of interest (GOI), 5′ and 3′ untranslated regions (UTRs), poly(A) tail. Each element can be modified or selected accordingly to improve the stability and translation of the mRNA, as has been thoroughly explored in other publications [86,87].

Most commonly, the T7 promoter sequence (5′-TAATACGACTCACTATA-3′) is used for recognition by the T7 RNA polymerase during IVT, considered the standard polymerase for manufacturing purposes [88]. If using the cap analog CleanCap AG, an additional A is required at the 3′ end of the promoter sequence for an AGG initiator sequence [89]. If the manufacturer aims to use a different RNA polymerase, such as from T3 or SP6, the corresponding promoter must be present in the DNA template. Additionally, in the case of a plasmid DNA construct, an antibiotic resistance marker sequence for bacterial selection and restriction sites for DNA template linearization are required.

In applications in which the gene of interest encodes for a therapeutic protein, optimization of the coding sequence can be performed to reduce protein immunogenicity and increase protein expression [90]. For instance, codon optimization can lead to more controllable translation and increased mRNA half-life [78,86,90,91]. It has been reported that high GC content increases mRNA stability, ribosome association, and thus translation efficiency [86,92,93]. Optimization of the GC content in the GOI, with concurrent uridine depletion in therapeutic mRNA design, not only improves the elongation rate and translation efficiency, but can also alter RNA secondary structures that can interfere with gene expression [78].

Both the 5′- and 3′-UTRs are indispensable for the stability and translation initiation of the therapeutic mRNA molecule being delivered [94]. The β-globin UTRs have been widely used in both clinical trials and research contexts [95,96]. 5′-UTR features such as the length, sequence elements and secondary structures play an important role in translation initiation during scanning [97], with the average length of 5′-UTR in eukaryotes ranging from ~100 to ~200 nucleotides (nt) in mammals [86]. However, it has been proposed that a shorter 5′-UTR with at least 20 nt minimizes the scanning process and thus maximizes protein expression [86]. Moreover, highly stable secondary structures near the 5′-end should be avoided as they can disrupt ribosome loading and scanning [98,99], and potential upstream start codons should be eliminated to avoid leaky scanning [86]. Selective translation can be achieved by introducing additional sequence elements to the 5′-UTR depending on the therapeutic purpose. In the context of cancer therapy, special 5′-UTR elements capable of translation under nutrient restriction may be needed for intratumor mRNA injection [86].

The 3′-UTR, similarly to the 5′-UTR, contains regulatory elements that affect translation efficiency and mRNA stability. It is generally believed that a shorter 3′-UTR increases the stability of the mRNA due to the loss of microRNA binding sites, thus escaping mRNA degradation [86]. Additionally, the use of two sequential β-globin 3′UTRs resulted in significantly higher maximum protein levels and prolonged persistence of the protein [100]. Lastly, high throughput techniques have been developed for 3′-UTR optimizations, including a novel cell-based selection process to identify 3′-UTRs that increase protein expression encoded by synthetic mRNA [101] and a massive parallel functional assay for optimization [102].

The poly(A) tail plays a key role in mRNA translation and stability, as it protects the mRNA from nuclease degradation [103]. The poly(A) tail can be added to the mRNA either by using a poly(A) polymerase after transcription or by already having a poly(A) sequence in the DNA template [3]. The latter is the standard practice for clinical applications, as it allows for a consistent, predetermined length of the poly(A) tail [104]. The extension of the poly(A) tail up to 120 nt has been demonstrated to improve translation efficiency, showing that the length is an important aspect to consider [105,106]. Additionally, Trepotec et al. demonstrated the benefit of using segmented poly(A) tails, which consists of at least two A-containing elements of 40–60 adenosines separated by a spacer element of different length. This segmented approach avoids recombination of plasmid DNA during bacterial replication without impairing protein expression and mRNA half-life [107].

### 3.2. Upstream Process: DNA Template Production

For use in IVT, a linearized and purified DNA template is required. Although there are several approaches for the manufacturing of the template DNA, the space is currently dominated by the use of bacterial fermentation to obtain plasmid DNA. Recently, synthetic and enzymatic approaches have also been proposed for the manufacturing of these templates, including PCR.

#### 3.2.1. Bacterial Fermentation Approach

Plasmid DNA generation is generally performed through fermentation of *Escherichia coli* (*E. coli*). Several different strains have been reported for pDNA production such as DH5α [108], DH5 [109], DH10B [110], DH1 [111], JM108 [112], and SCS1-L [113]. Among them, DH5α remains the standard strain used in laboratory and industry practices due to the existence of effective widespread protocols that have been previously established [112]. For this methodology, *E. coli* competent cells are transformed with the designed DNA plasmids. At the industrial scale, bacterial culture expansion follows three main steps: inoculation for the creation of a master cell bank, shake flask fermentation, and large-scale bioreactor fermentation. To improve pDNA production yields using this production platform, several strategies can be adopted such as selecting a high-producing bacterial strain and/or combining varying medium composition and culture strategies such as batch or fed-batch mode [114].

Vector engineering has contributed to higher yields such as the use of pUC-based plasmids and R1-based plasmids [114]. A study conducted by Lopes et al. demonstrated that a fed-batch mode culture leads to higher plasmid volumetric yields compared to a batch-mode culture [114]. Another study by Carnes et al. demonstrated that using standard high-copy pUC origin-containing plasmids and novel control parameters for fed-batch fermentation resulted in increased specific pDNA yield with respect to cell mass (up to 1500 mg/L of culture medium) compared to 100–250 mg/L for typical plasmid fermentation media and processes [115]. An *E. coli* DH5α culture in the fed-batch mode, with glucose and glycerol as initial carbon sources in the batch phase, showed a 2.2-fold increase compared to similar feeding phases but with no glycerol [114]. In fact, glycerol is considered a complementary carbon source of glucose because of its high specific plasmid DNA productivity and can be used to increase plasmid yield up to 70.6% [116]. The most used growth medium for *E. coli* is Luria Broth (LB) with yeast extract as the nitrogen source, although other medium compositions have successfully been used, including a modified MBL medium [116].

#### 3.2.2. Synthetic DNA Approach

To avoid the cloning and preparation steps involved in pDNA production through bacterial fermentation, which are both costly and time consuming, several publications and industrial production platforms have adopted alternative template production methods. The fermentation process can take several days or, at times, several weeks, and it involves expensive reagents, including bacteria and antibiotics [117]. The associated risk of biocontamination in the final product has also become a concern for the good manufacturing practice (GMP) of these therapeutics in the clinical industry [83]. As such, synthetic DNA approaches such as PCR, which allow for time-effective manufacturing, have been used to generate the DNA template for mRNA synthesis [3].

Traditional PCR has successfully been used to produce a DNA template for IVT [118]. In 2022, de Mey et al. introduced a novel approach for the production of mRNA based on a synthetic DNA template generated using assembly PCR with synthetic oligonucleotides [83]. It was reported that using this method, the DNA template amplification can go up to several micrograms, allowing for a fast transition from the DNA production step to the mRNA synthesis in only a few hours [83].

Other synthetic DNA production methods have also been described. For example, Touchlight Genetics Ltd. developed a synthetic DNA manufacturing platform using an in vitro dual enzyme process [117]. This proprietary enzymatic platform enables multi-gram DNA production in weeks, allowing for rapid and large-scale production [117]. However, despite the potential of synthetic DNA template production, these approaches have yet to be established for longer DNA strands. It is crucial to consider not only yield but also the error rate, as mutations should be avoided in the sequence. To produce RNA for therapeutic applications, DNA templates are required to be several kilobases long, and thus bacterial fermentation currently remains most appropriate for these productions.

### 3.3. Upstream Process: DNA Template Purification

For use in the IVT reaction, the DNA template must be purified and linearized to ensure the quality of the subsequently produced mRNA. The purification process is most extensive in the case of bacterial fermentation. Purification of pDNA from bacterial cells typically begins with an alkaline lysis step after cell harvesting [119,120], in which a detergent solution such as sodium dodecyl sulfate (SDS) and sodium hydroxide are used to disrupt the cell membrane [84]. Next, the lysate is neutralized before clarification [84,119,121]. However, due to the viscous nature of the resulting precipitate, separation of cellular components can only be performed by pre-filtration or centrifugation followed by clearing filtration, which can be time-consuming and expensive [122]. Moreover, DNA sensitivity to shear stress requires low shear stress techniques to gently mix the cell lysate and the neutralizing agent, such as a flotation-based method described in a patent by PlasmidFactory [121,122]. Another method that has been explored for pDNA purification is boiling cell lysis [123], which has been successfully scaled up using a streamlined method of plasmid DNA extraction by continual thermal lysis [124].

In many traditional plasmid DNA purification processes, RNAse enzymes are used to degrade the RNA prior to proceeding with chromatography steps for pDNA isolation [125]. However, RNAse A is purified from bovine components and is of concern in large scale manufacturing [125]. Furthermore, for a process ultimately aiming to produce RNA, the addition of RNAse should be avoided. To this end, RNAse-free purification methods have been proposed: Duval et al. implemented calcium chloride precipitation, followed by tangential flow filtration (TFF) for the removal of high molecular weight RNA and low molecular weight RNA species, respectively [125,126]. These two steps also contribute to the reduction in microbial proteins and chromosomal DNA and to concentration of the product. In addition, performing concentration prior to chromatography reduces column loading time, accelerating the overall process [126].

For pDNA isolation, chromatography steps based on three different principles result in high-purity plasmid free of host DNA, RNA, proteins, and endotoxins: size-exclusion chromatography, ion-exchange chromatography, and hydrophobic interaction chromatography [119,127,128,129,130]. The plasmids can be collected in several isoforms: supercoiled circular isoforms, open circular, and linear. A hydrophobic interaction chromatography method has been proposed to select for supercoiled pDNA, as it is the most stable isoform [130,131].

Finally, the DNA template must be linearized to prepare for mRNA transcription. For the use of the SP6 and T7 RNA polymerase during IVT, a 5′overhang is known to be preferable to ensure the stability of the polymerase and to reduce artifacts [132]. To achieve this overhang after linearization, endonucleases such as HindIII, SpeI, SapI, NotI, EcoRI may be used [133]. Removal of this enzyme as well as of the un-linearized DNA is required to isolate the final DNA template and proceed with transcription. In both laboratory- and industry-scale productions, phenol chloroform extraction has been established as the gold standard technique for this step. However, this technique requires the management of highly hazardous materials and is no longer preferred for large-scale clinical operations [14]. As such, Cui et al. demonstrated a method using positively charged resins for strong anion exchange chromatography, which was deemed comparable to phenol chloroform extraction in terms of the quality of the resulting mRNA [14]. Subsequent TFF allows for removal of smaller impurities while filtering the DNA template into an appropriate solvent for the subsequent IVT [133].

### 3.4. Upstream Process: mRNA Synthesis

#### 3.4.1. Enzymatic Synthesis

mRNA is produced by IVT, a relatively rapid and simple process in which an RNA polymerase consumes NTPs (nucleotide triphosphates) to catalyse the synthesis of the mRNA from the corresponding DNA linear template. The required components include RNA Polymerase, NTPs, Magnesium (MgCl_2_), and a reaction buffer. Various bacteriophage polymerases have been used in the field, such as T7, T3, or SP6 RNA polymerases [134]. The T7 RNA polymerase (T7 RNAP) is the most used RNAP in both research and industry, owing to its ability to produce full-length RNA transcripts (longer than 20 kb) with high fidelity [134,135].

Despite its high fidelity and tolerance for incorporation of non-natural NTPs [135], T7 RNAP can also generate immunostimulatory by-products such as double-stranded RNA (dsRNA) which may affect protein expression and render the downstream purification process more difficult [136,137]. Double-stranded RNA molecules are innate immune response activators and should therefore be avoided in therapeutic applications in which immunotolerance to the treatment is important [137,138,139]. The generation of dsRNA by-products can be significantly decreased by engineering a mutant T7 RNAP using computational, structural, mechanistic and laboratory screening approaches. For example, a double-mutant T7 RNAP (G47A + 884G) successfully reduced dsRNA content while maintaining RNA yield and purity [136]. Other advances include the development of thermostable RNA polymerases, such as Hi-T7 RNA Polymerase M0658 by BioLabs, which has been engineered to withstand IVT performed at high temperatures, preventing loopback transcription [140]. However, some experts have noted that high temperatures (≥48 °C) are difficult to scale up and may lead to RNA degradation [136]. Lastly, the addition of urea at a concentration of 1 M during IVT was shown to be an effective method to reduce the undesired nucleobase pairing that causes dsRNA formation [141].

Moreover, magnesium ions are required as a cofactor for the T7 polymerase. Kern et al. found that below 5 mM of free Mg^2+^, both the transcription rate and IVT efficiency are greatly reduced [142]. However, there is a lack of consensus on the ideal conditions for free Mg^2+^ concentration. While Sartorius claims that 12–20 mM of MgCl_2_ per reaction increases mRNA yield [143], Young et al. claim an ideal range between 50 and 60 mM of free Mg^2+^ [144]. Magnesium counter-ions also have an impact on mRNA yield, with both magnesium acetate and magnesium chloride having been successfully used for IVT [118,145]. A study showed that magnesium acetate is preferred over chloride [145], which was corroborated by Samnuan et al. [146].

The use of modified NTPs such as N1-methylpseudouridine (m1ψ) has been found to reduce the immunogenicity of synthetic mRNA and to drive high levels of protein production, which is in part attributed to its ability to blunt TLR3 activation [135]. The incorporation of other modified nucleotides such as pseudouridine (ψ), 5-methylcytidine (m5C), N6-methyladenosine (m6A), 5-methyluridine (m5U), or 2-thiouridine (s2U) have also shown reduced immunostimulatory effect of the delivered RNA and enhanced translation [135,147,148,149]. The reduction in immune stimulation and increase in stability of the mRNA molecule are especially important in the context of mRNA therapeutics and protein replacement therapies where degradation and lack of translation pose a direct obstacle to the function of the product.

The addition of spermidine to the reaction mixture at a concentration of 1 to 3 mM has been shown to enhance transcription while having an inhibitory effect at higher concentrations [118]. A design of experiment (DoE) performed by Samnuan et al. found that spermidine enhances transcription when present at a concentration of 0.2 to 2 mM [146], further supporting its use.

Mature mRNA requires the 5′ cap structure for mRNA stability and gene expression. A Cap 1 (m^7^GpppN_1_mp) structure is preferred for optimal mRNA stability and expression, as it is recognized as self by the immune system [150]. Cap 0 (m^7^GpppNp), on the other hand, can activate an innate immune response, impairing stability and expression levels [151]. There are two main methods for capping: (1) post-translational capping, where the transcribed mRNA is capped in an additional step using enzymes, or (2) co-transcriptional capping, a one-step process where a Cap analog is incorporated in the IVT reaction.

The vaccinia virus capping enzyme (VCE) is commonly used for enzymatic capping and results in a Cap 0 structure [152]. An additional step using 2′-O-methyltransferase (2′-O-MTase) modifies Cap 0 into a Cap 1 structure [153], reaching a capping efficiency of up to 100% [151]. Moderna has successfully employed this capping strategy in their mRNA-1273 vaccine against SARS-CoV-2 [154]. It is important to note, however, that the addition of several enzymatic steps, including added purification and buffer exchange between the steps, can make the process more difficult to streamline and control [151].

The second capping method uses cap analogs co-transcriptionally, thus reducing production steps. Early iterations of cap analogs had the risk of mRNA elongation in the reverse direction, reducing translation efficiency [155,156]. To avoid this, the anti-reverse cap analog (ARCA) emerged; however, it could only be used to generate constructs with a Cap 0 structure and lower capping efficiency (60–80%) [151,157]. Later, CleanCap technology revolutionized the field as this cap analog allowed for a co-transcriptional addition of the naturally occurring Cap 1 structure at 90–99% efficiency [151], which was successfully implemented in the Pfizer-BioNTech BNT162b2 vaccine against SARS-CoV-2 for emergency use [158]. Cap analogs can lead to a simpler and faster process compared to enzymatic capping, but the use of patented technology can come at a high cost, which should be carefully analyzed in comparison to the cost of enzymatic capping [8].

The IVT reaction may be conducted in both batch and fed-batch modes. The fed-batch mode involves the addition of NTPs and Mg feed during the reaction, as these components have the highest impact on the rate of the reaction as well as the yield [159]. This approach was first demonstrated in 1999 by Kern et al. to produce short RNA molecules [160]. Fed-batch IVT has since been employed on larger mRNA molecules to achieve increased yields [161]. The consumption of NTPs may be monitored using HPLC throughout the duration of the reaction, and they can be supplemented accordingly [159]. Both exceedingly low and high NTP concentrations have been shown to be limiting to the production of RNA, thus supporting the approach to control NTP levels throughout production [146]. This method has previously led to mRNA production with yields of up to 12 g/L [159] compared to the usual 5 g/L [19], showing the immense potential in process intensification.

#### 3.4.2. Towards Automated Production of mRNA

Although production of mRNA therapeutics in a continuous mode has yet to be implemented, several publications have explored this perspective [8,162,163,164]. The intensification of mRNA manufacturing by integrating production and purification in a continuous manner could decrease the hold times and freeze–thaw cycles during the process, potentially increasing the quality and yield of the final product. This could address the challenge in obtaining a higher amount of product with low immunogenicity required for the repeated dosing of mRNA therapeutics. Self-amplifying RNA (saRNA) has been proposed as a modality to reduce manufacturing burden and costs, as a lower dose is required to reach the same level of protein expression as conventional mRNA [19]. However, it is important to note that saRNA can have immunogenic effects [165] that would make it undesirable for chronically dosed treatments.

Continuous processes require the implementation of controls throughout the production to validate the product and to facilitate their automation. Well-defined process models are vital in the transition to continuous automated production. To this end, Helgers et al. produced in silico models for the continuous production of mRNA in plug flow and continuously stirred tank reactors and determined a theoretical improvement factor of 56 times for the space-time yield in comparison to batch production in a continuously stirred tank reactor [162]. Vetter et al. also suggested that the use of control loops, such as proportional integral derivative (PID) control, can be key to improving productivity, robustness and compliance with critical quality attributes (CQA) [163]. Rosa et al. and Ouranidis et al. produced digital designs and conceptual designs for the continuous end-to-end manufacturing of mRNA therapeutics, establishing the initial frameworks for future experimental work to build upon these initial approaches [8,164].

#### 3.4.3. Solid-Phase Synthesis

First established in 1963 by Merrifield for the production of peptides [166], the chemical synthesis method was adapted to the production of short oligonucleotides and advanced by Beaucage and Caruthers [167,168]. Once scaled up to the industrial scale, this method allowed for the production of oligonucleotides up to the kilogram scale, and it can be fully automated [169,170]. The method relies on phosphoramidite chemistry and involves the cyclical addition of nucleosides in a sequence-specific manner on a solid support [171,172]. However, this method for RNA production is only suitable for short oligomers, with some claiming its ability to form chains of up to 40 nucleotides and others using this method extending up to approximately 100–150 nucleotides [153,173,174]. This renders this method currently unsuitable for mRNA production, but rather, it is appropriate for the production of siRNA, miRNA and anti-sense oligonucleotide (ASO) molecules as a straightforward chemical synthesis. It is possible to generate longer RNA molecules through the synthesis of two separate strands and joining them together through ligation strategies, namely T4 ligase [175,176], but this has not yet reached the efficiency and productivity that is established with enzymatic synthesis of mRNA.

### 3.5. Downstream Process: mRNA Purification

Isolation of complete mRNA transcripts from reagents and reaction by-products is critical for both product functionality and regulatory considerations. Process-related impurities include residual reagents (DNA template, enzymes, unincorporated NTPs) and by-products (immunogenic dsRNA and aborted mRNA products). DNA, RNA (around 300 kDa per kb [177]) and T7 polymerase (99 kDa [14]) are the larger components, while NTPs and cap analogs are much smaller (less than 1 kDa). The DNA template is typically larger than the mRNA produced, as the linearized plasmid includes a backbone sequence that is not transcribed. The purification of mRNA is essential to ensure immunotolerance and to achieve biologically active and therapeutically administrable mRNA [18].

The removal of the DNA template is typically accomplished via enzymatic digestion with DNAse I prior to other purification steps [8,178,179], followed by inactivation with EDTA. However, industry experts have previously indicated that the use of DNAse I may lead to the small DNA template fragments hybridizing to the final mRNA product [133]. Alternatively, chromatography capture methods have been implemented in some processes to remove the DNA template without digestion. Oligo-dT purification is of particular interest due to its ability to bind the polyA tail of the complete mRNA transcripts, without binding the DNA template, truncated mRNA transcripts, unused nucleotides, and the enzyme [133,180]. Cui et al. demonstrated that Oligo-dT chromatography purification may lead to mRNA recoveries of over 90% [14]. However, Oligo-dT purification may not be sufficient to separate dsRNA from the product and must be followed by polishing chromatography steps in the cases of high dsRNA content [8]. Several other chromatography techniques have been demonstrated for mRNA purification, but they were typically preceded by a TFF step, while the Oligo-dT method was used directly as a capture step for a more streamlined process [14].

For therapeutic applications, an emphasis has been placed on the elimination of dsRNA from the IVT mixture to improve translation efficacy and limit induction of cytokines. Several strategies have been described in the literature to ease the burden on purification by avoiding dsRNA formation throughout the reaction, including the addition of urea or use of modified T7 RNAP, as discussed in the mRNA synthesis section. The gold standard purification method for the removal of dsRNA is high-performance liquid chromatography (HPLC) using an alkylated non-porous polystyrene-divinylbenzene copolymer matrix [181]. A simple method using cellulose has proven successful to selectively remove dsRNA at the small scale [182]. Other methods, such as hydroxyapatite chromatography, core bead chromatography, and anion exchange chromatography, have been explored to improve the scalability of the platform [14]. For example, GSK has previously detailed an RNA purification method using DNAse, TFF, and CaptoCore resins, a core bead chromatography resin [183]. These purification methods may be product dependent; however, it has previously been demonstrated that mRNA recovery using core bead chromatography varies based on the length of the sequence [14].

In summary, multiple scalable chromatography methods have been explored for mRNA purification, with further efforts being made to streamline the process while maintaining a high quality of the product. For large-scale mRNA manufacturing, methods which require heating the mRNA sample or using organic solvents should preferably be avoided [14]. It is crucial that the chosen process ensures low levels of immunostimulatory by-products for chronically dosed therapeutic approaches [151].

### 3.6. Downstream Process: mRNA Delivery

The delivery of naked mRNA into in vivo models was first performed in 1990 by Wolff et al., demonstrating the feasibility of the technique [1]. Though the delivery of naked RNA has since been conducted in animal models and in clinical trials, these RNAs are unstable and can be easily degraded by ribonuclease enzymes during administration [184,185,186]. Thompson et al. found that higher concentrations of RNA are required to overcome the degradation and clearance of naked RNA in plasma, with over 90% of the RNA being cleared within 30 min of administration [187]. In addition, the size, negative charge, and hydrophilicity of messenger RNA molecules hinder their diffusion across the cell membrane [188]. Phua et al. evaluated the transfection efficiency of naked mRNA through several modes of administration and observed rapidly decreasing expression of the naked mRNA when delivered intravenously or intranasally [184]. This instability has previously been presented as an advantage for the safety profile of these therapeutics, but it nonetheless presents a challenge in their transfection efficiency and their long-term storage. To prevent degradation and facilitate cellular entry for cell and gene therapy applications, mRNA therapeutic delivery systems have been developed. Lipid-based and polymeric delivery of mRNA allowed this technology a successful entrance to the clinical space, with lipid nanoparticles (LNPs) being the most prevalent.

Lipid-based delivery particles have also received a considerable amount of attention in the clinical space, with liposomes being the first FDA approved lipid-based nanocarriers in 1990 [189]. Further progress was achieved when another class of lipid-based nanocarriers, lipid nanoparticles (LNPs), were efficient in RNA delivery in non-human primates [190,191]. Finally, the success of these carriers was exemplified during the SARS-CoV-2 pandemic, with both the BioNTech and Moderna vaccines utilizing LNP technology for the delivery of mRNA vaccines [5]. Furthermore, the expansion of lipid libraries and lipid-like compounds over the past two decades has allowed for the identification of novel lipid nanocarrier compositions for RNA delivery [192].

Liposomes are composed of a singular lipid bilayer with an aqueous core, whereas the lipid nanoparticle contains several lipid layers and microdomains of lipids and the oligonucleotide [193]. Liposomes for nucleic acid delivery range between 20 and 1000 nanometers (nm) in size and are generally composed of a cationic lipid along with stabilizers such as cholesterol [188]. In 1987, the formation of cationic liposomes for nucleic acid delivery was first demonstrated [194]. Despite their success in vitro, permanently charged liposomes were unsuccessful in the clinical space due to toxicity, and neutral lipids were inefficient at encapsulating nucleic acids [195]. The emergence of the ionizable cationic lipid, which acquires a positive charge according to the surrounding pH, surmounted this issue and led to the creation of ionizable LNPs [196].

LNPs first entered the therapeutic space with the approval of Onpattro, an siRNA drug product, in 2018 [2]. The vectors are primarily composed of an ionizable cationic lipid, and are supplemented by helper lipids, polyethylene glycol (PEG)-lipids and cholesterol [197]. Ionizable cationic lipids acquire a positive charge according to the surrounding pH, and thus promote interaction and complex formation with the mRNA [195]. For immunotherapeutic applications, studies have demonstrated that the ionizable lipid chosen should have a pKa value between 6.6 and 6.9 in order to elicit an appropriate immune response [198]. The helper lipids, which are commonly phospholipids, and cholesterol serve to stabilize the nanoparticle and aid in the endosomal escape of the mRNA [199]. PEG-lipids reduce aggregation of the LNPs and prolong the circulation time of the particles once administered. The molar ratio at which the lipid components are mixed is not uniform across mRNA therapeutic products and may impact the biological activity of the drug product. Roces et al. demonstrated that altering the lipid molar ratios impacts both size and zeta potential of the LNPs [200]. These characteristics of the LNP impact the biodistribution and immunogenicity of the drug product and should therefore be optimized according to the application. Studies evaluating lipid-based systems administered through subcutaneous and intramuscular injection have shown clear effects of the size on their uptake [201,202]. A study originating from Moderna demonstrated that LNP sizes between 60 and 150 nm were found to produce strong immune responses in non-human primates [201]. Thus, immunotherapeutic mRNA LNP products are likely to be produced within this range [201].

As previously established, administration of LNPs through intravenous and intramuscular methods leads to non-specific accumulation of the particles in the liver [203]. Consequently, this accumulation may lead to side effects of the vaccine including hepatic inflammation and hepatic necrosis [204], and could limit access for patients with preexisting inflammatory conditions [205]. The ability to redirect mRNA LNP therapeutics to their target tissue is therefore critical in order to reduce off-target effects [206]. Further work to improve the distribution of LNPs to target-specific tissues is ongoing [207]. Most prominently, selective organ targeting (SORT) nanoparticles, developed by Cheng et al., demonstrated that supplementing currently established LNP compositions with specified percentages of charged molecules can alter tissue tropism [208]. Furthermore, the spleen has been shown to be effectively targeted through the use of mRNA coated lipoplexes by adjusting the charge ratio (lipid to RNA) to 1.3:2 [209]. Coating strategies for mRNA LNPs have also been explored [210], including new avenues being PEG coatings for redirection of other nanoparticles from the liver to the target tissue [211,212]. These technologies may reduce dose requirements for mRNA therapeutics intended for expression in specific tissues due to their ability to direct the LNPs rather than their non-specific biodistribution.

#### 3.6.1. Microfluidic Manufacturing of mRNA-LNPs

The lipid concentration, the mRNA concentration, the lipid to mRNA ratio as well as the encapsulation technique impact the particle size, polydispersity, surface charge and RNA encapsulation efficiency, all of which are critical quality attributes of the product and impact the activity of the product [213]. As such, the encapsulation protocol should be carefully optimized to meet these standards.

mRNA LNPs are most commonly formulated using T-junction mixing and microfluidic mixing, with work ongoing to improve the scalability of these processes for clinical applications. At a laboratory scale, sonication and bulk mixing have been used, but they present limitations in reproducibility and scalability [214]. Benchtop instruments such as Precision Nanosystems Spark and Ignite systems have been adopted for mRNA LNP formation, both in laboratories and in the industry. There are, however, a number of publications which have designed custom microfluidic devices for this formulation step.

Table 2 summarizes microfluidic mixers which have been used for the encapsulation of RNA into lipid-based delivery systems. Optimization of the flow rates and flow rate ratios (FRR) for each of the devices are uniquely tailored to the device and have not been generalized across microfluidic designs. However, overall trends concerning the operating parameter have been observed in several publications. For example, Roces et al. confirmed that an increase in overall flow rate results in a decrease in size as well as increases in the FRR [200]. These results were corroborated by Gkionis et al. using a different microfluidic system [215].

#### 3.6.2. mRNA-LNP Formulation

Thermostability and physical stability of mRNA-LNPs remains a challenge in the translation of mRNA therapeutics to the market. In non-urgent applications, as in the case of therapeutic use rather than the case of pandemic use, the product may require long-term storage prior to its use. Furthermore, to improve treatment accessibility and transport, refrigerated or room temperature storage are preferable. Both lyophilized and liquid formulations have been considered for the improvement of mRNA-LNP storage. It has been demonstrated that the choice of buffer and cryopreservatives impact the stability of the LNPs both in liquid and solid formulations. Henderson et al. found that, for example, the Hepes buffer better maintains the morphology of the LNPs after freeze–thaw compared to Tris and PBS [224]. Furthermore, their study found that LNPs stored in Tris buffer lead to improved expression of the delivered gene compared to those stored in PBS and Hepes [224]. Zhao et al. evaluated cryopreservatives in the long-term storage of lipid-like nanoparticles under aqueous, freezing and lyophilized conditions [225]. Their results suggested that, when stored at 4 °C, the particles lose the majority of their delivery efficiency within 5 months of storage. Additionally, they found that sucrose and trehalose outperformed mannitol maintaining the delivery efficiency when freezing in liquid nitrogen [225]. Similar results were obtained concerning the efficacy of both sucrose and trehalose at preserving LNPs throughout freezing for LNPs encapsulating siRNA [226]. Similarly, Kim et al. found that LNPs stored in 10% *w*/*v* sucrose in PBS did not lose potency after 1 month of storage. Despite most of the current publications presenting data in support of freezing of mRNA-LNPs rather than aqueous conditions, Zhang et al. previously presented a thermostable aqueous vaccine which remains stable for 7 days at 4 °C and 25 °C [227].

Furthermore, formulations undoubtedly vary based on the target application and administration method as well. For example, for lung delivery through inhalation, the formulation requires nebulization. This process can easily destabilize the mRNA-LNPs, impacting size, encapsulation efficiency and subsequent mRNA expression. While the lipid composition of the LNPs impacts their stability throughout the aerosolization process [228], formulation excipients can also be used to decrease changes in these critical quality attributes throughout the process. A patent from Moderna, for example, has previously described mRNA-LNP formulation variations appropriate for nebulization which include the addition of P188 and sucrose to a Tris buffer [54]. Their formulations were also able to maintain both size and encapsulation efficiency over the course of 19 freeze–thaw cycles [54].

Several studies involving the lyophilization of mRNA-LNPs have also been completed since the evolution of the lipid nanoparticle [229,230,231]. Lyophilization promises the ability to store mRNA-LNP formulations at room temperature without the effects of hydrolysis, as is the case of aqueous formulations [229]. Lamoot et al. found that the addition of 20% sucrose (*w*/*v*) in a Tris-based buffer allowed for successful lyophilization without major impacts on LNP size, zeta potential or in vitro expression of the encoded gene [229]. Additionally, Muramatsu et al. found that lyophilization of mRNA LNPs in the presence of sucrose (10% *w*/*v*) and maltose (10% *w*/*v*) provided long-term stability (for at least 12 weeks) of the formulation both at room temperature and at 4 °C [229]. These results emphasize the need for the evaluation of cryoprotectants and lypoprotectants throughout the manufacturing process of lipid-based mRNA therapeutics.

## 4. Conclusions and Future Perspectives

The revolution of mRNA technology in vaccination highlighted the potential of mRNA therapeutic development, with over 80 ongoing clinical trials and immense interest from companies. The diverse scope of applications for this powerful tool continue to expand, ranging from gene editing to cancer immunotherapy and protein replacement therapy. Additionally, the technology continues to be further refined as new techniques are studied for tissue-targeting with the idea of tailored UTRs [86] and tissue-specific miRNA control systems [232] for selective mRNA translation, as well as development of nanoparticles for targeted delivery [208]. Innovations are also underway in administration methods, with the convenience of the inhalation route and the specificity of intratumoral injections incentivizing further studies.

However, challenges remain when it comes to manufacturing and commercializing this novel class of therapeutics. The regulatory framework for a drug modality with such diverse applications remains unclear, and further streamlining of the approval process could speed up commercialization of new mRNA products [17]. Classification of RNA-based therapies as gene therapies in the literature is unclear due to the fact that RNA therapeutics do not necessarily lead to modification of the patients’ genetic material [3]. While some literature does not distinguish between RNA therapy and gene therapy [103,233,234,235], others strictly refer to gene silencing and gene delivery via RNA molecules as RNA therapeutics [236]. It is therefore clear that as the number of applications and trials for mRNA-based therapeutics grows, harmonization of these definitions is required across regulatory agencies to establish the necessary requirements and controls for the manufacturing of these products, allowing for a more streamlined roll-out of new mRNA therapeutic products. Manufacturers have to closely monitor how such regulatory requirements and definitions evolve to ensure more effective development and data generation for their product.

Moreover, therapeutics have different dose requirements when compared to vaccines, and thus pose different manufacturing scale requirements. The scale can be as small as hospital-based production for personalized medicine [29], and as extensive as large-scale production for a supply of milligram doses administered multiple times a month. A greater focus on process intensification, especially automated and tightly controlled continuous manufacturing, can lead to higher yields and improved product quality, as well as reducing the dependence on the bottleneck of costly GMP-grade reagents. It is also important to emphasize formulation suitable for long-term storage of mRNA, with further studies of both solid and liquid formulations, as therapeutic drugs are not distributed and administered with the same urgency as vaccines during a pandemic.

Additionally, immunotolerance is a crucial characteristic for many chronically dosed treatments [4]; thus, it is essential to tightly control the levels of immunostimulatory by-products like dsRNA by avoiding its formation [136,140,141] or removing it with an adequate purification pipeline [14,182]. Further progress in synthetic approaches to producing DNA template and long RNA molecules could alleviate the burden on purification and revolutionize the mRNA manufacturing landscape. Overall, as production methods become more well-established and suitable for all these purposes, standardization of mRNA manufacturing in the industry can facilitate regulatory approval and enable consistent product yield and quality.

## Figures and Tables

**Table 1:** Lipid Carrier-Based mRNA Therapeutics in Ongoing Clinical Trials as of August 2023.

Trial ID	Status	Indication	Treatment Name	Dose Regimen	Administration Method
NCT04573140	Recruiting	Adult glioblastoma	Autologous total tumor mRNA and pp65 full-length (fl) lysosomal-associated membrane protein (LAMP) mRNA-loaded DOTAP liposome	Every 2 weeks (3 cycles), monthly (15 cycles)	IV
NCT05097911	Recruiting	Advanced Hepatocellular Carcinoma	MTL-CEBPA	Day 1 and Day 8 of a 21-Day Dosing Schedule	IV
NCT05579275	Recruiting	Advanced malignant solid tumors	JCXH-212 Injection	Every 3 weeks (up to 8 cycles)	Unspecified injection
NCT05949775	Not yet recruiting	Advanced Malignant Solid Tumours	Neoantigen mRNA Personalized Cancer vaccine	Every 3 weeks (9 cycles)	SQ
NCT05978102	Not yet recruiting	Advanced Solid Tumor	STI-7349/IL2v mRNA	Every 3 weeks	IV
NCT05533697	Recruiting	Advanced Solid Tumours	mRNA- 4359	N/A	IM
NCT02872025	Recruiting	Carcinoma, Intraductal, Noninfiltrating	mRNA 2752	2–4 Doses	ILES
NCT05659264	Recruiting	Chronic heart failure	mRNA-0184	2 groups: single dose OR 4 doses every 16 weeks	IV
NCT05141721	Recruiting	Colorectal neoplasms	GRT-R902 (samRNA), GRT-C901(viral vector)	4 doses over first year	IM
NCT05712538	Recruiting	Cystic Fibrosis	ARCT-032	Single dose	INH
NCT05668741	Recruiting	Cystic Fibrosis	VX-522	Single dose	INH
NCT05938387	Recruiting	Glioblastoma	CV09050101 mRNA vaccine	7 doses at different intervals	IM
NCT05095727	Recruiting	Glycogen storage disease	mRNA-3745	Single dose. Additional dosages after >21 days	IV
NCT05497453	Recruiting	Hepatocellular Carcinoma	OTX-2002	At least 2 doses	IV
NCT04710641	Recruiting	Hepatocellular Carcinoma	MTL-CEBPA (saRNA)	Every 3 weeks	IV
NCT05120830	Recruiting	Hereditary Angioedema	NTLA-2002	Single dose	IV
NCT05933577	Recruiting	High-Risk Melanoma	V940	Every 3 weeks (up to 9 doses)	IM
NCT05295433	Recruiting	Isolated methylmalonic acidemia (MMA)	mRNA-3705	Every 2–4 weeks	IV
NCT04899310	Recruiting	Isolated methylmalonic acidemia (MMA)	mRNA-3705	Every 2–4 weeks	IV
NCT03289962	Active, not recruiting	Locally or Advanced Metastatic Cancer	RO7198457	Every 2 weeks	IV
NCT05969041	Recruiting	Malignant Epithelial Tumours	MT-302 (A)	Weekly–biweekly doses for first 3 doses. Every 4 weeks subsequently	IV
NCT05539157	Active, not recruiting	Malignant solid tumours, etc.	JCXH-211	Every 4 weeks (up to 3 doses)	IT
NCT05714748	Recruiting	Malignant Tumours	EBV mRNA vaccine	Weekly (4 doses), followed by a 1-month interval (1 dose)	IM
NCT03897881	Active	Melanoma	mRNA-4157	Every 3 weeks (up to 9 doses)	IM
NCT05264974	Not yet recruiting	Melanoma	Autologous total tumor mRNA loaded DOTAP liposome vaccine	Every 2 weeks	IV
NCT04526899	Recruiting	Melanoma	BNT111	N/A	IV
NCT03871348	Active, not recruiting	Metastatic Neoplasm	SAR441000	N/A	IT
NCT05142189	Recruiting	Non-Small Cell Lung Cancer	BNT116	N/A	IV
NCT04442347	Active, not recruiting	Ornithine Transcarbamylase Deficiency	ARCT-810	Single dose	IV
NCT05526066	Recruiting	Ornithine Transcarbamylase Deficiency	ARCT-810	Every 2 weeks (up to 6 doses)	IV
NCT04161755	Active, not recruiting	Pancreatic cancer	RO7198457	Every week (8 Cycles)	IV
NCT03953235	Active, not recruiting	Personalized cancer vaccine for many cancer types	GRT-R904 (samRNA), GRT-C903 (adenoviral vector)	N/A	N/A
NCT05130437	Recruiting	Propionic Acidemia	mRNA-3927	Every 3 weeks	IV
NCT04159103	Recruiting	Propionic Acidemia	mRNA-3927	Every 3 weeks (up to 10 doses)	IV
NCT04382898	Recruiting	Prostate Cancer	BNT112	N/A	IV
NCT05660408	Not yet recruiting	Pulmonary osteosarcoma	RNA-LP vaccine	Every 2 weeks (2 cycles), monthly (12 cycles)	N/A
NCT03739931	Recruiting	Relapsed solid tumor malignancies/lymphoma	mRNA-2752	Every 2 weeks	IT
NCT04503278	Recruiting	Solid Tumor	BNT211- CLDN6 CAR-T/CLDN6 CAR-T(A), CLDN6 RNA-LPX	N/A	IV
NCT05262530	Recruiting	Solid Tumor	BNT142	N/A	IV
NCT04710043	Recruiting	Solid Tumor	BNT152/BNT153	N/A	IV
NCT04455620	Recruiting	Solid Tumor	BNT151	N/A	IV
NCT03313778	Active, not recruiting	Solid tumours	mRNA-4157	9 cycles (once every 3 weeks)	IM
NCT03946800	Active, not recruiting	Solid tumours	MEDI1191	Every 3 weeks	IT
NCT04683939	Recruiting	Solid tumours, etc.	BNT141	N/A	IV
NCT04601051	Recruiting	Transthyretin-Related (ATTR) Familial Amyloid Polyneuropathy	NTLA-2001	Single dose	IV
NCT04534205	Recruiting	Unresectable Head and Neck Squamous Cell Carcinoma	BNT113	N/A	IV

IV: intravenous, IT: intratumoral, IM: intramuscular, ILES: intralesional, SQ: subcutaneous, INH: inhalation, N/A: Not Available.

**Table 2:** Microfluidic Designs in Literature for Encapsulation of RNA into Lipid-Based Delivery systems. Q total refers to the total flow rate.

Microfluidic Architecture	Channel Width	Flow Rate Operation	References
Ring Micromixer (Precision Nanosystems)	150–280 µm channel widths	Q total = 0.4 mL/min–20 mL/min	[216]
Staggered Herringbone Mixers	150 µm channel width	Q total = 0.024 mL/min–2.4 mL/min	[217]
Y junction entry with baffle mixer (iLiNP)	200 µm channel width	Q total = 0.05–0.5 mL/min	[218]
Staggered herringbone mixer with Y junction entry	200 µm channel width	Q total = 1 mL/min–5 mL/min	[219]
Staggered Herringbone mixer	200 µm channel width	Q total = 0.4 mL/min–1 mL/min	[220]
Staggered Herringbone mixer with Y junction entry	200 µm channel width	Q total = 0.02 to 4 mL/min	[221]
Baffle Mixer with Y junction entry	200 µm channel width	Q total = 0.05 mL/min	[222]
Hydrodynamic flow focusing	150 µm channel width	Q total = 0.20006–0.8001 mL/min	[215]
Spiral Mixing	300 µm channel width	Q total = 0.00666 mL/min	[223]

## References

[1] Wolff J.A., Malone R.W., Williams P., Chong W., Acsadi G., Jani A., Felgner P.L. (1990). Direct gene transfer into mouse muscle in vivo. Science.

[2] Kulkarni J.A., Witzigmann D., Chen S., Cullis P.R., van der Meel R. (2019). Lipid Nanoparticle Technology for Clinical Translation of siRNA Therapeutics. Acc. Chem. Res..

[3] Sahin U., Karikó K., Türeci Ö. (2014). mRNA-based therapeutics—Developing a new class of drugs. Nat. Rev. Drug Discov..

[4] Rohner E., Yang R., Foo K.S., Goedel A., Chien K.R. (2022). Unlocking the promise of mRNA therapeutics. Nat. Biotechnol..

[5] Jain S., Venkataraman A., Wechsler M.E., Peppas N.A. (2021). Messenger RNA-based vaccines: Past, present, and future directions in the context of the COVID-19 pandemic. Adv. Drug Deliv. Rev..

[6] Anderson E.J., Rouphael N.G., Widge A.T., Jackson L.A., Roberts P.C., Makhene M., Chappell J.D., Denison M.R., Stevens L.J., Pruijssers A.J. (2020). Safety and immunogenicity of SARS-CoV-2 mRNA-1273 vaccine in older adults. N. Engl. J. Med..

[7] Baden L.R., El Sahly H.M., Essink B., Kotloff K., Frey S., Novak R., Diemert D., Spector S.A., Rouphael N., Creech C.B. (2021). Efficacy and safety of the mRNA-1273 SARS-CoV-2 vaccine. N. Engl. J. Med..

[8] Rosa S.S., Prazeres D.M., Azevedo A.M., Marques M.P. (2021). mRNA vaccines manufacturing: Challenges and bottlenecks. Vaccine.

[9] Dammes N., Peer D. (2020). Paving the Road for RNA Therapeutics. Trends Pharmacol. Sci..

[10] Geall A.J., Ulmer J.B. (2015). Introduction to RNA-based vaccines and therapeutics. Expert Rev. Vaccines.

[11] Maruggi G., Zhang C., Li J., Ulmer J.B., Yu D. (2019). mRNA as a transformative technology for vaccine development to control infectious diseases. Mol. Ther..

[12] Moço P.D., Xu X., Silva C.A.T., Kamen A.A. (2023). Production of adeno-associated viral vector serotype 6 by triple transfection of suspension HEK293 cells at higher cell densities. Biotechnol. J..

[13] Pascolo S. (2017). Messenger RNA: The inexpensive biopharmaceutical. J. Multidiscip. Eng. Sci. Technol..

[14] Cui T., Fakhfakh K., Turney H., Güler-Gane G., Toloczko A., Hulley M., Turner R. (2023). Comprehensive studies on building a scalable downstream process for mRNAs to enable mRNA therapeutics. Biotechnol. Prog..

[15] Leung A.K., Tam Y.Y.C., Cullis P.R. (2014). Lipid nanoparticles for short interfering RNA delivery. Adv. Genet..

[16] American Society of Cell and Gene Therapy (2023). Gene, Cell, & RNA Therapy Landscape; Q1 2023 Quarterly Data Report.

[17] Vervaeke P., Borgos S., Sanders N., Combes F. (2022). Regulatory guidelines and preclinical tools to study the biodistribution of RNA therapeutics. Adv. Drug Deliv. Rev..

[18] Webb C., Ip S., Bathula N.V., Popova P., Soriano S.K., Ly H.H., Eryilmaz B., Nguyen Huu V.A., Broadhead R., Rabel M. (2022). Current status and future perspectives on MRNA drug manufacturing. Mol. Pharm..

[19] Kis Z., Kontoravdi C., Shattock R., Shah N. (2020). Resources, production scales and time required for producing RNA vaccines for the global pandemic demand. Vaccines.

[20] Michel T., Wendel H.-P., Krajewski S. (2016). Next-generation therapeutics: mRNA as a novel therapeutic option for single-gene disorders. Mod. Tools Genet. Eng..

[21] Ramaswamy S., Tonnu N., Tachikawa K., Limphong P., Vega J.B., Karmali P.P., Chivukula P., Verma I.M. (2017). Systemic delivery of factor IX messenger RNA for protein replacement therapy. Proc. Natl. Acad. Sci. USA.

[22] An D., Schneller J.L., Frassetto A., Liang S., Zhu X., Park J.-S., Theisen M., Hong S.-J., Zhou J., Rajendran R. (2017). Systemic messenger RNA therapy as a treatment for methylmalonic acidemia. Cell Rep..

[23] Magadum A., Kaur K., Zangi L. (2019). mRNA-based protein replacement therapy for the heart. Mol. Ther..

[24] Zangi L., Lui K.O., Von Gise A., Ma Q., Ebina W., Ptaszek L.M., Später D., Xu H., Tabebordbar M., Gorbatov R. (2013). Modified mRNA directs the fate of heart progenitor cells and induces vascular regeneration after myocardial infarction. Nat. Biotechnol..

[25] Zhang X., Men K., Zhang Y., Zhang R., Yang L., Duan X. (2019). Local and systemic delivery of mRNA encoding survivin-T34A by lipoplex for efficient colon cancer gene therapy. Int. J. Nanomed..

[26] Martini P.G., Guey L.T. (2019). A new era for rare genetic diseases: Messenger RNA therapy. Hum. Gene Ther..

[27] Córdoba K.M., Jericó D., Sampedro A., Jiang L., Iraburu M.J., Martini P.G., Berraondo P., Avila M.A., Fontanellas A. (2022). Messenger RNA as a personalized therapy: The moment of truth for rare metabolic diseases. Int. Rev. Cell Mol. Biol..

[28] Kulkarni J.A., Cullis P.R., Van Der Meel R. (2018). Lipid nanoparticles enabling gene therapies: From concepts to clinical utility. Nucleic Acid Ther..

[29] Damase T.R., Sukhovershin R., Zhang M., Kiss D.L., Cooke J.P. (2022). Hospital-based RNA Therapeutics. Messenger RNA Therapeutics.

[30] Burris H.A., Patel M.R., Cho D.C., Clarke J.M., Gutierrez M., Zaks T.Z., Frederick J., Hopson K., Mody K., Binanti-Berube A. (2019). A phase I multicenter study to assess the safety, tolerability, and immunogenicity of mRNA-4157 alone in patients with resected solid tumors and in combination with pembrolizumab in patients with unresectable solid tumors. J. Clin. Oncol..

[31] Damase T.R., Sukhovershin R., Boada C., Taraballi F., Pettigrew R.I., Cooke J.P. (2021). The limitless future of RNA therapeutics. Front. Bioeng. Biotechnol..

[32] Zhang H.-X., Zhang Y., Yin H. (2019). Genome editing with mRNA encoding ZFN, TALEN, and Cas9. Mol. Ther..

[33] Foster J.B., Barrett D.M., Karikó K. (2019). The emerging role of in vitro-transcribed mRNA in adoptive T cell immunotherapy. Mol. Ther..

[34] Musunuru K., Chadwick A.C., Mizoguchi T., Garcia S.P., DeNizio J.E., Reiss C.W., Wang K., Iyer S., Dutta C., Clendaniel V. (2021). In vivo CRISPR base editing of PCSK9 durably lowers cholesterol in primates. Nature.

[35] Intellia Therapeutics (2020). Study to Evaluate Safety, Tolerability, Pharmacokinetics, and Pharmacodynamics of NTLA-2001 in Patients With Hereditary Transthyretin Amyloidosis with Polyneuropathy (ATTRv-PN) and Patients with Transthyretin Amyloidosis-Related Cardiomyopathy (ATTR-CM).

[36] Intellia Therapeutics (2021). NTLA-2002 in Adults With Hereditary Angioedema (HAE).

[37] ModernaTX, Inc (2021). A Long-Term Extension Study to Evaluate the Safety and Clinical Activity of mRNA-3927.

[38] ModernaTX, Inc. (2022). A Study of mRNA-3745 in Adult and Pediatric Participants with Glycogen Storage Disease Type 1a (GSD1a).

[39] ModernaTX, Inc. (2021). Open-Label Study of mRNA-3927 in Participants With Propionic Acidemia.

[40] ModernaTX, Inc., Merck Sharp & Dohme LLC (2019). An Efficacy Study of Adjuvant Treatment with the Personalized Cancer Vaccine mRNA-4157 and Pembrolizumab in Participants with High-Risk Melanoma (KEYNOTE-942).

[41] Memorial Sloan Kettering Cancer Center, Genentech, Inc. (2019). Study of Personalized Tumor Vaccines (PCVs) and a PD-L1 Blocker in Patients With Pancreatic Cancer That Can be Treated with Surgery.

[42] Gritstone bio, Inc. (2019). ; Squibb, B.-M. A Study of a Personalized Cancer Vaccine Targeting Shared Neoantigens.

[43] Stemirna Therapeutics, Peking University Cancer Hospital & Institute (2023). Clinical Study of mRNA Vaccine in Patients with Advanced Malignant Solid Tumors.

[44] BioNTech SE (2022). Safety, Pharmacokinetics, Pharmacodynamics, and Preliminary Efficacy Trial of BNT141 in Patients with Unresectable or Metastatic CLDN18.2-Positive Gastric, Pancreatic, Ovarian and Biliary Tract Tumors.

[45] BioNTech SE (2022). Safety and Preliminary Efficacy Trial of BNT142 in Patients with CLDN6-Positive Solid Tumors.

[46] Nitika, Wei J., Hui A.-M. (2022). The delivery of mRNA vaccines for therapeutics. Life.

[47] Dilliard S.A., Cheng Q., Siegwart D.J. (2021). On the mechanism of tissue-specific mRNA delivery by selective organ targeting nanoparticles. Proc. Natl. Acad. Sci. USA.

[48] Patel M., Jimeno A., Wang D., Stemmer S., Bauer T., Sweis R., Geva R., Kummar S., Reagan P., Perets R. (2021). 539 Phase 1 study of mRNA-2752, a lipid nanoparticle encapsulating mRNAs encoding human OX40L/IL-23/IL-36γ, for intratumoral (ITu) injection+/-durvalumab in advanced solid tumors and lymphoma. Sci. Transl. Med..

[49] The Fourth Affiliated Hospital of Zhejiang University School of Medicine (2023). A Dose Escalation and Dose Expansion Clinical Study of STI-7349 in Subjects with Advanced Solid Tumors.

[50] Longhurst H., Fijen L., Lindsay K., Butler J., Golden A., Maag D., Xu Y., Cohn D. (2022). In vivo CRISPR/Cas9 editing of KLKB1 in patients with Hereditary Angioedema: A First-in-Human Study. Ann. Allergy Asthma Immunol..

[51] Cui T., Li B., Li W. (2022). NTLA-2001: Opening a new era for gene therapy. Life Med..

[52] Carneiro B.A., Zamarin D., Marron T., Mehmi I., Patel S.P., Subbiah V., El-Khoueiry A., Grand D., Garcia-Reyes K., Goel S. (2022). Abstract CT183: First-in-human study of MEDI1191 (mRNA encoding IL-12) plus durvalumab in patients (pts) with advanced solid tumors. Cancer Res..

[53] Roh E.H., Fromen C.A., Sullivan M.O. (2022). Inhalable mRNA vaccines for respiratory diseases: A roadmap. Curr. Opin. Biotechnol..

[54] Mike Smith O.A., Brito L. (2017). Stabilized Formulations of Lipid Nanoparticles. U.S. Patent.

[55] Vertex Pharmaceuticals Incorporated (2023). A Phase 1 Study of VX-522 in Participants with Cystic Fibrosis (CF).

[56] Arcturus Therapeutics, Inc., Novotech CRO (2023). Safety, Tolerability, and Pharmacokinetics of ARCT-032 in Healthy Adult Subjects.

[57] Oslo University Hospital (2010). Vaccine Therapy in Curative Resected Prostate Cancer Patients.

[58] Oslo University Hospital (2018). Dendritic Cell Immunotherapy Against Cancer Stem Cells in Glioblastoma Patients Receiving Standard Therapy.

[59] Berneman Z., Kanker K.O.T., Kanker S., Flanders R.F. (2012). ; Antwerp University Hospital. Efficacy Study of Dendritic Cell Vaccination in Patients with Acute Myeloid Leukemia in Remission.

[60] Kanker K.O.T., Semmy S., Antwerp University Hospital, Olivia Hendrickx Research Fund vzw (2021). Adjuvant Dendritic Cell Immunotherapy for Pediatric Patients With High-Grade Glioma or Diffuse Intrinsic Pontine Glioma.

[61] Kanker K.O.T., Kanker S., Antwerp University Hospital (2017). Autologous Dendritic Cell Vaccination in Mesothelioma.

[62] Antwerp University Hospital (2015). Adjuvant Dendritic Cell-immunotherapy Plus Temozolomide in Glioblastoma Patients.

[63] Affiliated Hospital to Academy of Military Medical Sciences (2021). Clinical Study of DC-AML Cells in the Treatment of Acute Myeloid Leukemia. Identifier NCT05000801..

[64] Sholler G. (2021). PEACH TRIAL- Precision Medicine and Adoptive Cellular Therapy.

[65] Immunomic Therapeutics, Inc., University of Florida, National Cancer Institute (2016). Vaccine Therapy for the Treatment of Newly Diagnosed Glioblastoma Multiforme.

[66] University of Florida, Immunomic Therapeutics, Inc. (2022). RENEW: Feasibility of CMV RNA-Pulsed Dendritic Cells Vaccines for the Treatment of Newly Diagnosed Glioblastoma Patients.

[67] CoImmune (2020). Dendritic Cell Immunotherapy Plus Standard Treatment of Advanced Renal Cell Carcinoma.

[68] Lion TCR Pte. Ltd. (2022). Redirected HBV-Specific T Cells in Patients with HBV-Related HCC (SAFE-T-HBV).

[69] Lion TCR Pte. Ltd. (2022). Study of HBV-TCR T Cells (LioCyx-M) as Monotherapy or as Combination with Lenvatinib for HBV-Related HCC.

[70] Ruijin Hospital, UTC Therapeutics Inc (2021). Anti-Mesothelin CAR-T Cells With Advanced Refractory Solid Tumors.

[71] CytoMed Therapeutics Pte Ltd., National University Hospital (2022). Allogeneic NKG2DL-Targeting CAR γδ T Cells (CTM-N2D) in Advanced Cancers.

[72] 2Seventy Bio Inc. A Study of bbT369 in Relapsed and/or Refractory B Cell Non-Hodgkin’s Lymphoma (NHL). Identifier NCT05169489 2022..

[73] Cartesian Therapeutics (2019). Descartes-11 in Multiple Myeloma.

[74] Cartesian Therapeutics (2019). Descartes-08 CAR-T Cells in Generalized Myasthenia Gravis (MG).

[75] Lyerly H., Merck Sharp & Dohme LLC, Duke University (2019). A Study to Evaluate Concurrent VRP-HER2 Vaccination and Pembrolizumab for Patients with Breast Cancer. Identifier NCT03632941..

[76] Tang-Du Hospital, Air Force Military Medical University (2021). Exosome-Based Nanoplatform for Ldlr mRNA Delivery in FH.

[77] Youn H., Chung J.-K. (2015). Modified mRNA as an alternative to plasmid DNA (pDNA) for transcript replacement and vaccination therapy. Expert Opin. Biol. Ther..

[78] Mauger D.M., Cabral B.J., Presnyak V., Su S.V., Reid D.W., Goodman B., Link K., Khatwani N., Reynders J., Moore M.J. (2019). mRNA structure regulates protein expression through changes in functional half-life. Proc. Natl. Acad. Sci. USA.

[79] Wei H.-H., Zheng L., Wang Z. (2023). mRNA therapeutics: New vaccination and beyond. Fundam. Res..

[80] Huang X., Kong N., Zhang X., Cao Y., Langer R., Tao W. (2022). The landscape of mRNA nanomedicine. Nat. Med..

[81] Liu X., Zhang Y., Zhou S., Dain L., Mei L., Zhu G. (2022). Circular RNA: An emerging frontier in RNA therapeutic targets, RNA therapeutics, and mRNA vaccines. J. Control. Release.

[82] Marques R., Lacerda R., Romão L. (2022). Internal Ribosome Entry Site (IRES)-Mediated Translation and Its Potential for Novel mRNA-Based Therapy Development. Biomedicines.

[83] de Mey W., De Schrijver P., Autaers D., Pfitzer L., Fant B., Locy H., Esprit A., Lybaert L., Bogaert C., Verdonck M. (2022). A synthetic DNA template for fast manufacturing of versatile single epitope mRNA. Mol. Ther.-Nucleic Acids.

[84] Prather K.J., Sagar S., Murphy J., Chartrain M. (2003). Industrial scale production of plasmid DNA for vaccine and gene therapy: Plasmid design, production, and purification. Enzym. Microb. Technol..

[85] Deng Z., Tian Y., Song J., An G., Yang P. (2022). mRNA vaccines: The dawn of a new era of cancer immunotherapy. Front. Immunol..

[86] Jia L., Qian S.-B. (2021). Therapeutic mRNA Engineering from Head to Tail. Acc. Chem. Res..

[87] Xia X. (2021). Detailed Dissection and Critical Evaluation of the Pfizer/BioNTech and Moderna mRNA Vaccines. Vaccines.

[88] Trepotec Z., Aneja M.K., Geiger J., Hasenpusch G., Plank C., Rudolph C. (2019). Maximizing the translational yield of mRNA therapeutics by minimizing 5′-UTRs. Tissue Eng. Part A.

[89] TriLink Biotechnologies CleanCap Reagent AG Product Insert (Catalog No. N-7113 Version v3). https://www.trilinkbiotech.com/media/folio3/productattachments/product_insert/n7113_insert_v3.pdf.

[90] Weng Y., Li C., Yang T., Hu B., Zhang M., Guo S., Xiao H., Liang X.-J., Huang Y. (2020). The challenge and prospect of mRNA therapeutics landscape. Biotechnol. Adv..

[91] McClellan D.A. (2000). The codon-degeneracy model of molecular evolution. J. Mol. Evol..

[92] Mordstein C., Savisaar R., Young R.S., Bazile J., Talmane L., Luft J., Liss M., Taylor M.S., Hurst L.D., Kudla G. (2020). Codon usage and splicing jointly influence mRNA localization. Cell Syst..

[93] Thess A., Grund S., Mui B.L., Hope M.J., Baumhof P., Fotin-Mleczek M., Schlake T. (2015). Sequence-engineered mRNA without chemical nucleoside modifications enables an effective protein therapy in large animals. Mol. Ther..

[94] Qin S., Tang X., Chen Y., Chen K., Fan N., Xiao W., Zheng Q., Li G., Teng Y., Wu M. (2022). mRNA-based therapeutics: Powerful and versatile tools to combat diseases. Signal Transduct. Target. Ther..

[95] Kariko K., Kuo A., Barnathan E. (1999). Overexpression of urokinase receptor in mammalian cells following administration of the in vitro transcribed encoding mRNA. Gene Ther..

[96] Benteyn D., Anguille S., Van Lint S., Heirman C., Van Nuffel A.M., Corthals J., Ochsenreither S., Waelput W., Van Beneden K., Breckpot K. (2013). Design of an optimized Wilms’ tumor 1 (WT1) mRNA construct for enhanced WT1 expression and improved immunogenicity in vitro and in vivo. Mol. Ther. Nucleic Acids.

[97] Leppek K., Das R., Barna M. (2018). Author Correction: Functional 5′ UTR mRNA structures in eukaryotic translation regulation and how to find them. Nat. Rev. Mol. Cell Biol..

[98] Ding Y., Tang Y., Kwok C.K., Zhang Y., Bevilacqua P.C., Assmann S.M. (2014). In vivo genome-wide profiling of RNA secondary structure reveals novel regulatory features. Nature.

[99] Wan Y., Qu K., Zhang Q.C., Flynn R.A., Manor O., Ouyang Z., Zhang J., Spitale R.C., Snyder M.P., Segal E. (2014). Landscape and variation of RNA secondary structure across the human transcriptome. Nature.

[100] Holtkamp S., Kreiter S., Selmi A., Simon P., Koslowski M., Huber C., Tureci O.z., Sahin U. (2006). Modification of antigen-encoding RNA increases stability, translational efficacy, and T-cell stimulatory capacity of dendritic cells. Blood.

[101] von Niessen A.G.O., Poleganov M.A., Rechner C., Plaschke A., Kranz L.M., Fesser S., Diken M., Löwer M., Vallazza B., Beissert T. (2019). Improving mRNA-based therapeutic gene delivery by expression-augmenting 3′ UTRs identified by cellular library screening. Mol. Ther..

[102] Zhao W., Pollack J.L., Blagev D.P., Zaitlen N., McManus M.T., Erle D.J. (2014). Massively parallel functional annotation of 3′ untranslated regions. Nat. Biotechnol..

[103] Kwon H., Kim M., Seo Y., Moon Y.S., Lee H.J., Lee K., Lee H. (2018). Emergence of synthetic mRNA: In vitro synthesis of mRNA and its applications in regenerative medicine. Biomaterials.

[104] Kim S.C., Sekhon S.S., Shin W.-R., Ahn G., Cho B.-K., Ahn J.-Y., Kim Y.-H. (2021). Modifications of mRNA vaccine structural elements for improving mRNA stability and translation efficiency. Mol. Cell. Toxicol..

[105] Kormann M.S., Hasenpusch G., Aneja M.K., Nica G., Flemmer A.W., Herber-Jonat S., Huppmann M., Mays L.E., Illenyi M., Schams A. (2011). Expression of therapeutic proteins after delivery of chemically modified mRNA in mice. Nat. Biotechnol..

[106] Jalkanen A.L., Coleman S.J., Wilusz J. (2014). Determinants and implications of mRNA poly (A) tail size–does this protein make my tail look big?. Semin. Cell Dev. Biol..

[107] Trepotec Z., Geiger J., Plank C., Aneja M.K., Rudolph C. (2019). Segmented poly(A) tails significantly reduce recombination of plasmid DNA without affecting mRNA translation efficiency or half-life. RNA.

[108] Lara A.R., Knabben I., Regestein L., Sassi J., Caspeta L., Ramírez O.T., Büchs J. (2011). Comparison of oxygen enriched air vs. pressure cultivations to increase oxygen transfer and to scale-up plasmid DNA production fermentations. Eng. Life Sci..

[109] Listner K., Bentley L., Okonkowski J., Kistler C., Wnek R., Caparoni A., Junker B., Robinson D., Salmon P., Chartrain M. (2006). Development of a highly productive and scalable plasmid DNA production platform. Biotechnol. Prog..

[110] Lahijani R., Hulley G., Soriano G., Horn N.A., Marquet M. (1996). High-yield production of pBR322-derived plasmids intended for human gene therapy by employing a temperature-controllable point mutation. Hum. Gene Ther..

[111] Cooke J.R., McKie E.A., Ward J.M., Keshavarz-Moore E. (2004). Impact of intrinsic DNA structure on processing of plasmids for gene therapy and DNA vaccines. J. Biotechnol..

[112] Selas Castiñeiras T., Williams S.G., Hitchcock A.G., Smith D.C. (2018). *E. coli* strain engineering for the production of advanced biopharmaceutical products. FEMS Microbiol. Lett..

[113] Singer A., Eiteman M.A., Altman E. (2009). DNA plasmid production in different host strains of Escherichia coli. J. Ind. Microbiol. Biotechnol..

[114] Lopes M.B., Gonçalves G.A., Felício-Silva D., Prather K.L., Monteiro G.A., Prazeres D.M., Calado C.R. (2015). In situ NIR spectroscopy monitoring of plasmid production processes: Effect of producing strain, medium composition and the cultivation strategy. J. Chem. Technol. Biotechnol..

[115] Carnes A.E., Hodgson C.P., Williams J.A. (2006). Inducible Escherichia coli fermentation for increased plasmid DNA production. Biotechnol. Appl. Biochem..

[116] Xu Z.-N., Shen W.-H., Chen H., Cen P.-L. (2005). Effects of medium composition on the production of plasmid DNA vector potentially for human gene therapy. J. Zhejiang Univ. SCIENCE B.

[117] Ohlson J. (2020). Plasmid manufacture is the bottleneck of the genetic medicine revolution. Drug Discov. Today.

[118] Rosa S.S., Nunes D., Antunes L., Prazeres D.M., Marques M.P., Azevedo A.M. (2022). Maximizing mRNA vaccine production with Bayesian optimization. Biotechnol. Bioeng..

[119] Sun B., Yu X., Yin Y., Liu X., Wu Y., Chen Y., Zhang X., Jiang C., Kong W. (2013). Large-scale purification of pharmaceutical-grade plasmid DNA using tangential flow filtration and multi-step chromatography. J. Biosci. Bioeng..

[120] Bimboim H.C., Doly J. (1979). A rapid alkaline extraction procedure for screening recombinant plasmid DNA. Nucleic Acids Res..

[121] Voss C., Flaschel E. (2010). Method for Producing Extra-Chromosomal Nucleic Acid Molecules. U.S. Patent.

[122] Schmeer M., Schleef M. (2014). Pharmaceutical grade large-scale plasmid DNA manufacturing process. DNA Vaccines: Methods and Protocols.

[123] Holmes D.S., Quigley M. (1981). A rapid boiling method for the preparation of bacterial plasmids. Anal. Biochem..

[124] Zhu K., Jin H., He Z., Zhu Q., Wang B. (2006). A continuous method for the large-scale extraction of plasmid DNA by modified boiling lysis. Nat. Protoc..

[125] Eon-Duval A., Gumbs K., Ellett C. (2003). Precipitation of RNA impurities with high salt in a plasmid DNA purification process: Use of experimental design to determine reaction conditions. Biotechnol. Bioeng..

[126] Eon-Duval A., MacDuff R.H., Fisher C.A., Harris M.J., Brook C. (2003). Removal of RNA impurities by tangential flow filtration in an RNase-free plasmid DNA purification process. Anal. Biochem..

[127] Latulippe D.R., Zydney A.L. (2009). Size exclusion chromatography of plasmid DNA isoforms. J. Chromatogr. A.

[128] Eon-Duval A., Burke G. (2004). Purification of pharmaceutical-grade plasmid DNA by anion-exchange chromatography in an RNase-free process. J. Chromatogr. B.

[129] Bo H., Wang J., Chen Q., Shen H., Wu F., Shao H., Huang S. (2013). Using a single hydrophobic-interaction chromatography to purify pharmaceutical-grade supercoiled plasmid DNA from other isoforms. Pharm. Biol..

[130] Černigoj U., Štrancar A. (2021). Scale-up of plasmid DNA downstream process based on chromatographic monoliths. DNA Vaccines: Methods and Protocols.

[131] Parker T., Cherradi Y., Mishra N. (2020). Scalable Purification of Plasmid DNA: Strategies and Considerations for Vaccine and Gene Therapy Manufacturing.

[132] Hornblower B., Robb G.B., Tzertzinis G. Minding Your Caps and Tails—Considerations for Functional mRNA Synthesis. https://international.neb.com/tools-and-resources/feature-articles/minding-your-caps-and-tails.

[133] Bancel S., Issa W.J., Aunins J.G., Chakraborty T. (2018). Manufacturing Methods for Production of RNA Transcripts. U.S. Patent.

[134] Kwon S., Kwon M., Im S., Lee K., Lee H. (2022). mRNA vaccines: The most recent clinical applications of synthetic mRNA. Arch. Pharmacal Res..

[135] Nance K.D., Meier J.L. (2021). Modifications in an emergency: The role of N1-methylpseudouridine in COVID-19 vaccines. ACS Cent. Sci..

[136] Dousis A., Ravichandran K., Hobert E.M., Moore M.J., Rabideau A.E. (2023). An engineered T7 RNA polymerase that produces mRNA free of immunostimulatory byproducts. Nat. Biotechnol..

[137] Mu X., Greenwald E., Ahmad S., Hur S. (2018). An origin of the immunogenicity of in vitro transcribed RNA. Nucleic Acids Res..

[138] Durbin A.F., Wang C., Marcotrigiano J., Gehrke L. (2016). RNAs containing modified nucleotides fail to trigger RIG-I conformational changes for innate immune signaling. mBio.

[139] Peisley A., Jo M.H., Lin C., Wu B., Orme-Johnson M., Walz T., Hohng S., Hur S. (2012). Kinetic mechanism for viral dsRNA length discrimination by MDA5 filaments. Proc. Natl. Acad. Sci. USA.

[140] Wu M.Z., Asahara H., Tzertzinis G., Roy B. (2020). Synthesis of low immunogenicity RNA with high-temperature in vitro transcription. RNA.

[141] Piao X., Yadav V., Wang E., Chang W., Tau L., Lindenmuth B.E., Wang S.X. (2022). Double-stranded RNA reduction by chaotropic agents during in vitro transcription of messenger RNA. Mol. Ther. Nucleic Acids.

[142] Kern J.A., Davis R.H. (1997). Application of Solution Equilibrium Analysis to inVitro RNA Transcription. Biotechnol. Prog..

[143] Nemec K.S., Livk A.G., Celjar A.M., Skok J., Sekirnik R., Kostelec T., Gagnon P., Štrancar A. (2021). Effect of Mg^2+^ Ion Concentration on IVT Reaction Kinetics Determined by Novel Rapid Analytical HPLC Assay.

[144] Young J.S., Ramirez W.F., Davis R.H. (1997). Modeling and optimization of a batch process for in vitro RNA production. Biotechnol. Bioeng..

[145] Nikolic M., Gasiūnienė M., Asa D., Šeputienė V. (2022). Determination of the Optimal Buffer Conditions and Nucleotide Concentrations to Maximize mRNA Yield Using In Vitro Transcription.

[146] Samnuan K., Blakney A.K., McKay P.F., Shattock R.J. (2021). Design-of-experiments in vitro transcription yield optimization of self-amplifying RNA. bioRxiv.

[147] Karikó K., Muramatsu H., Welsh F.A., Ludwig J., Kato H., Akira S., Weissman D. (2008). Incorporation of pseudouridine into mRNA yields superior nonimmunogenic vector with increased translational capacity and biological stability. Mol. Ther..

[148] Fotin-Mleczek M., Duchardt K.M., Lorenz C., Pfeiffer R., Ojkic-Zrna S., Probst J., Kallen K.-J. (2011). Messenger RNA-based vaccines with dual activity induce balanced TLR-7 dependent adaptive immune responses and provide antitumor activity. J. Immunother..

[149] Yang L., Tang L., Zhang M., Liu C. (2022). Recent advances in the molecular design and delivery technology of mRNA for vaccination against infectious diseases. Front. Immunol..

[150] Borden K.L. (2016). The eukaryotic translation initiation factor eIF4E wears a “cap” for many occasions. Translation.

[151] Linares-Fernández S., Lacroix C., Exposito J.-Y., Verrier B. (2020). Tailoring mRNA vaccine to balance innate/adaptive immune response. Trends Mol. Med..

[152] Fang E., Liu X., Li M., Zhang Z., Song L., Zhu B., Wu X., Liu J., Zhao D., Li Y. (2022). Advances in COVID-19 mRNA vaccine development. Signal Transduct. Target. Ther..

[153] Pradère U., Halloy F., Hall J. (2017). Chemical synthesis of long RNAs with terminal 5′-phosphate groups. Chem. Eur. J..

[154] Corbett K.S., Edwards D.K., Leist S.R., Abiona O.M., Boyoglu-Barnum S., Gillespie R.A., Himansu S., Schäfer A., Ziwawo C.T., Di Piazza A.T. (2020). SARS-CoV-2 mRNA vaccine design enabled by prototype pathogen preparedness. Nature.

[155] Jemielity J., Fowler T., Zuberek J., Stepinski J., Lewdorowicz M., Niedzwiecka A., Stolarski R., Darzynkiewicz E., Rhoads R.E. (2003). Novel “anti-reverse” cap analogs with superior translational properties. RNA.

[156] Stepinski J., Waddell C., Stolarski R., Darzynkiewicz E., Rhoads R.E. (2001). Synthesis and properties of mRNAs containing the novel “anti-reverse” cap analogs 7-methyl (3′-O-methyl) GpppG and 7-methyl (3′-deoxy) GpppG. RNA.

[157] Kuhn A., Diken M., Kreiter S., Selmi A., Kowalska J., Jemielity J., Darzynkiewicz E., Huber C., Türeci Ö., Sahin U. (2010). Phosphorothioate cap analogs increase stability and translational efficiency of RNA vaccines in immature dendritic cells and induce superior immune responses in vivo. Gene Ther..

[158] Henderson J.M., Ujita A., Hill E., Yousif-Rosales S., Smith C., Ko N., McReynolds T., Cabral C.R., Escamilla-Powers J.R., Houston M.E. (2021). Cap 1 messenger RNA synthesis with co-transcriptional cleancap^®^ analog by in vitro transcription. Curr. Protoc..

[159] Pregeljc D., Skok J., Vodopivec T., Mencin N., Krušič A., Ličen J., Nemec K.Š., Štrancar A., Sekirnik R. (2023). Increasing yield of in vitro transcription reaction with at-line high pressure liquid chromatography monitoring. Biotechnol. Bioeng..

[160] Kern J.A., Davis R.H. (1999). Application of a fed-batch system to produce RNA by in vitro transcription. Biotechnol. Prog..

[161] Skok J., Megušar P., Vodopivec T., Pregeljc D., Mencin N., Korenč M., Krušič A., Celjar A.M., Pavlin N., Krušič J. (2022). Gram-Scale mRNA Production Using a 250-mL Single-Use Bioreactor. Chem. Ing. Tech..

[162] Helgers H., Hengelbrock A., Schmidt A., Strube J. (2021). Digital twins for continuous mRNA production. Processes.

[163] Vetter F.L., Zobel-Roos S., Mota J.P.B., Nilsson B., Schmidt A., Strube J. (2022). Toward Autonomous Production of mRNA-Therapeutics in the Light of Advanced Process Control and Traditional Control Strategies for Chromatography. Processes.

[164] Ouranidis A., Davidopoulou C., Tashi R.-K., Kachrimanis K. (2021). Pharma 4.0 continuous mRNA drug products manufacturing. Pharmaceutics.

[165] Liu C., Shi Q., Huang X., Koo S., Kong N., Tao W. (2023). mRNA-based cancer therapeutics. Nat. Rev. Cancer.

[166] Merrifield R.B. (1963). Solid phase peptide synthesis. I. The synthesis of a tetrapeptide. J. Am. Chem. Soc..

[167] Beaucage S., Caruthers M. (1981). Deoxynucleoside phosphoramidites—A new class of key intermediates for deoxypolynucleotide synthesis. Tetrahedron Lett..

[168] RL L., Mahadevan V. (1965). Oligonucleotide synthesis on a polymer support. J. Am. Chem. Soc..

[169] Li N.-S., Frederiksen J.K., Piccirilli J.A. (2012). Automated solid-phase synthesis of RNA oligonucleotides containing a nonbridging phosphorodithioate linkage via phosphorothioamidites. J. Org. Chem..

[170] Sanghvi Y.S. (2019). Large-scale automated synthesis of therapeutic oligonucleotides: A status update. Adv. Nucleic Acid Ther..

[171] Cedillo I., Chreng D., Engle E., Chen L., McPherson A.K., Rodriguez A.A. (2017). Synthesis of 5′-GalNAc-conjugated oligonucleotides: A comparison of solid and solution-phase conjugation strategies. Molecules.

[172] Kumar R.K., Guzaev A.P., Rentel C., Ravikumar V.T. (2006). Efficient synthesis of antisense phosphorothioate oligonucleotides using a universal solid support. Tetrahedron.

[173] Ryczek M., Pluta M., Błaszczyk L., Kiliszek A. (2022). Overview of Methods for Large-Scale RNA Synthesis. Appl. Sci..

[174] Flamme M., McKenzie L.K., Sarac I., Hollenstein M. (2019). Chemical methods for the modification of RNA. Methods.

[175] Yu C.-H., Kabza A.M., Sczepanski J.T. (2021). Assembly of long l-RNA by native RNA ligation. Chem. Commun..

[176] Kershaw C.J., O’Keefe R.T. (2012). Splint ligation of RNA with T4 DNA ligase. Recombinant and In Vitro RNA Synthesis: Methods and Protocols.

[177] Kowalski P.S., Rudra A., Miao L., Anderson D.G. (2019). Delivering the messenger: Advances in technologies for therapeutic mRNA delivery. Mol. Ther..

[178] Whitley J., Zwolinski C., Denis C., Maughan M., Hayles L., Clarke D., Snare M., Liao H., Chiou S., Marmura T. (2022). Development of mRNA manufacturing for vaccines and therapeutics: mRNA platform requirements and development of a scalable production process to support early phase clinical trials. Transl. Res..

[179] Ouranidis A., Vavilis T., Mandala E., Davidopoulou C., Stamoula E., Markopoulou C.K., Karagianni A., Kachrimanis K. (2021). mRNA therapeutic modalities design, formulation and manufacturing under pharma 4.0 principles. Biomedicines.

[180] Von Der Mülbe F., Reidel L., Ketterer T., Gontcharova L., Bauer S., Pascolo S., Probst J., Schmid A. Method for Producing RNA. U.S. Patent US1001.

[181] Karikó K., Muramatsu H., Ludwig J., Weissman D. (2011). Generating the optimal mRNA for therapy: HPLC purification eliminates immune activation and improves translation of nucleoside-modified, protein-encoding mRNA. Nucleic Acids Res..

[182] Baiersdörfer M., Boros G., Muramatsu H., Mahiny A., Vlatkovic I., Sahin U., Karikó K. (2019). A facile method for the removal of dsRNA contaminant from in vitro-transcribed mRNA. Mol. Ther.-Nucleic Acids.

[183] Scorza F.B., Wen Y., Geall A., Porter F. (2016). RNA Purification Methods. U.S. Patent.

[184] Phua K.K., Leong K.W., Nair S.K. (2013). Transfection efficiency and transgene expression kinetics of mRNA delivered in naked and nanoparticle format. J. Control. Release.

[185] Sultana N., Magadum A., Hadas Y., Kondrat J., Singh N., Youssef E., Calderon D., Chepurko E., Dubois N., Hajjar R.J. (2017). Optimizing cardiac delivery of modified mRNA. Mol. Ther..

[186] Sahin U., Derhovanessian E., Miller M., Kloke B.-P., Simon P., Löwer M., Bukur V., Tadmor A.D., Luxemburger U., Schrörs B. (2017). Personalized RNA mutanome vaccines mobilize poly-specific therapeutic immunity against cancer. Nature.

[187] Thompson J.D., Kornbrust D.J., Foy J.W., Solano E.C., Schneider D.J., Feinstein E., Molitoris B.A., Erlich S. (2012). Toxicological and pharmacokinetic properties of chemically modified siRNAs targeting p53 RNA following intravenous administration. Nucleic Acid Ther..

[188] Tenchov R., Bird R., Curtze A.E., Zhou Q. (2021). Lipid nanoparticles─ from liposomes to mRNA vaccine delivery, a landscape of research diversity and advancement. ACS Nano.

[189] Thi T.T.H., Suys E.J., Lee J.S., Nguyen D.H., Park K.D., Truong N.P. (2021). Lipid-based nanoparticles in the clinic and clinical trials: From cancer nanomedicine to COVID-19 vaccines. Vaccines.

[190] Zimmermann T.S., Lee A.C., Akinc A., Bramlage B., Bumcrot D., Fedoruk M.N., Harborth J., Heyes J.A., Jeffs L.B., John M. (2006). RNAi-mediated gene silencing in non-human primates. Nature.

[191] Frank-Kamenetsky M., Grefhorst A., Anderson N.N., Racie T.S., Bramlage B., Akinc A., Butler D., Charisse K., Dorkin R., Fan Y. (2008). Therapeutic RNAi targeting PCSK9 acutely lowers plasma cholesterol in rodents and LDL cholesterol in nonhuman primates. Proc. Natl. Acad. Sci. USA.

[192] Akinc A., Zumbuehl A., Goldberg M., Leshchiner E.S., Busini V., Hossain N., Bacallado S.A., Nguyen D.N., Fuller J., Alvarez R. (2008). A combinatorial library of lipid-like materials for delivery of RNAi therapeutics. Nat. Biotechnol..

[193] Paunovska K., Loughrey D., Dahlman J.E. (2022). Drug delivery systems for RNA therapeutics. Nat. Rev. Genet..

[194] Felgner P.L., Gadek T.R., Holm M., Roman R., Chan H.W., Wenz M., Northrop J.P., Ringold G.M., Danielsen M. (1987). Lipofection: A highly efficient, lipid-mediated DNA-transfection procedure. Proc. Natl. Acad. Sci. USA.

[195] Schlich M., Palomba R., Costabile G., Mizrahy S., Pannuzzo M., Peer D., Decuzzi P. (2021). Cytosolic delivery of nucleic acids: The case of ionizable lipid nanoparticles. Bioeng. Transl. Med..

[196] Semple S.C., Klimuk S.K., Harasym T.O., Dos Santos N., Ansell S.M., Wong K.F., Maurer N., Stark H., Cullis P.R., Hope M.J. (2001). Efficient encapsulation of antisense oligonucleotides in lipid vesicles using ionizable aminolipids: Formation of novel small multilamellar vesicle structures. Biochim. Biophys. Acta (BBA)-Biomembr..

[197] Aldosari B.N., Alfagih I.M., Almurshedi A.S. (2021). Lipid nanoparticles as delivery systems for RNA-based vaccines. Pharmaceutics.

[198] Hassett K.J., Benenato K.E., Jacquinet E., Lee A., Woods A., Yuzhakov O., Himansu S., Deterling J., Geilich B.M., Ketova T. (2019). Optimization of lipid nanoparticles for intramuscular administration of mRNA vaccines. Mol. Ther. Nucleic Acids.

[199] Oberli M.A., Reichmuth A.M., Dorkin J.R., Mitchell M.J., Fenton O.S., Jaklenec A., Anderson D.G., Langer R., Blankschtein D. (2017). Lipid nanoparticle assisted mRNA delivery for potent cancer immunotherapy. Nano Lett..

[200] Roces C.B., Lou G., Jain N., Abraham S., Thomas A., Halbert G.W., Perrie Y. (2020). Manufacturing considerations for the development of lipid nanoparticles using microfluidics. Pharmaceutics.

[201] Hassett K.J., Higgins J., Woods A., Levy B., Xia Y., Hsiao C.J., Acosta E., Almarsson Ö., Moore M.J., Brito L.A. (2021). Impact of lipid nanoparticle size on mRNA vaccine immunogenicity. J. Control. Release.

[202] Oussoren C., Zuidema J., Crommelin D.J.A., Storm G. (1997). Lymphatic uptake and biodistribution of liposomes after subcutaneous injection.: II. Influence of liposomal size, lipid composition and lipid dose. Biochim. Biophys. Acta (BBA)-Biomembr..

[203] Pardi N., Tuyishime S., Muramatsu H., Kariko K., Mui B.L., Tam Y.K., Madden T.D., Hope M.J., Weissman D. (2015). Expression kinetics of nucleoside-modified mRNA delivered in lipid nanoparticles to mice by various routes. J. Control. Release.

[204] Chen J., Ye Z., Huang C., Qiu M., Song D., Li Y., Xu Q. (2022). Lipid nanoparticle-mediated lymph node–targeting delivery of mRNA cancer vaccine elicits robust CD8+ T cell response. Proc. Natl. Acad. Sci. USA.

[205] Parhiz H., Brenner J.S., Patel P.N., Papp T.E., Shahnawaz H., Li Q., Shi R., Zamora M.E., Yadegari A., Marcos-Contreras O.A. (2022). Added to pre-existing inflammation, mRNA-lipid nanoparticles induce inflammation exacerbation (IE). J. Control. Release.

[206] Anjaneyulu Dirisala J.L., Gonzalez-Carter D., Wang Z. (2023). Editorial: Delivery systems in biologics-based therapeutics. Front. Bioeng. Biotechnol..

[207] Żak M.M., Zangi L. (2021). Lipid nanoparticles for organ-specific mRNA therapeutic delivery. Pharmaceutics.

[208] Cheng Q., Wei T., Farbiak L., Johnson L.T., Dilliard S.A., Siegwart D.J. (2020). Selective organ targeting (SORT) nanoparticles for tissue-specific mRNA delivery and CRISPR–Cas gene editing. Nat. Nanotechnol..

[209] Kranz L.M., Diken M., Haas H., Kreiter S., Loquai C., Reuter K.C., Meng M., Fritz D., Vascotto F., Hefesha H. (2016). Systemic RNA delivery to dendritic cells exploits antiviral defence for cancer immunotherapy. Nature.

[210] Andretto V., Repellin M., Pujol M., Almouazen E., Sidi-Boumedine J., Granjon T., Zhang H., Remaut K., Jordheim L.P., Briançon S. (2023). Hybrid core-shell particles for mRNA systemic delivery. J. Control. Release.

[211] Dirisala A., Uchida S., Toh K., Li J., Osawa S., Tockary T.A., Liu X., Abbasi S., Hayashi K., Mochida Y. (2020). Transient stealth coating of liver sinusoidal wall by anchoring two-armed PEG for retargeting nanomedicines. Sci. Adv..

[212] Ouyang B., Poon W., Zhang Y.-N., Lin Z.P., Kingston B.R., Tavares A.J., Zhang Y., Chen J., Valic M.S., Syed A.M. (2020). The dose threshold for nanoparticle tumour delivery. Nat. Mater..

[213] Daniel S., Kis Z., Kontoravdi C., Shah N. (2022). Quality by Design for enabling RNA platform production processes. Trends Biotechnol..

[214] Wang X., Liu S., Sun Y., Yu X., Lee S.M., Cheng Q., Wei T., Gong J., Robinson J., Zhang D. (2023). Preparation of selective organ-targeting (SORT) lipid nanoparticles (LNPs) using multiple technical methods for tissue-specific mRNA delivery. Nat. Protoc..

[215] Gkionis L., Campbell R.A., Aojula H., Harris L.K., Tirella A. (2020). Manufacturing drug co-loaded liposomal formulations targeting breast cancer: Influence of preparative method on liposomes characteristics and in vitro toxicity. Int. J. Pharm..

[216] Ripoll M., Martin E., Enot M., Robbe O., Rapisarda C., Nicolai M.-C., Deliot A., Tabeling P., Authelin J.-R., Nakach M. (2022). Optimal self-assembly of lipid nanoparticles (LNP) in a ring micromixer. Sci. Rep..

[217] Shepherd S.J., Warzecha C.C., Yadavali S., El-Mayta R., Alameh M.-G., Wang L., Weissman D., Wilson J.M., Issadore D., Mitchell M.J. (2021). Scalable mRNA and siRNA lipid nanoparticle production using a parallelized microfluidic device. Nano Lett..

[218] Kimura N., Maeki M., Sato Y., Note Y., Ishida A., Tani H., Harashima H., Tokeshi M. (2018). Development of the iLiNP device: Fine tuning the lipid nanoparticle size within 10 nm for drug delivery. ACS Omega.

[219] Zhigaltsev I.V., Belliveau N., Hafez I., Leung A.K., Huft J., Hansen C., Cullis P.R. (2012). Bottom-up design and synthesis of limit size lipid nanoparticle systems with aqueous and triglyceride cores using millisecond microfluidic mixing. Langmuir.

[220] Maeki M., Saito T., Sato Y., Yasui T., Kaji N., Ishida A., Tani H., Baba Y., Harashima H., Tokeshi M. (2015). A strategy for synthesis of lipid nanoparticles using microfluidic devices with a mixer structure. RSC Adv..

[221] Belliveau N.M., Huft J., Lin P.J., Chen S., Leung A.K., Leaver T.J., Wild A.W., Lee J.B., Taylor R.J., Tam Y.K. (2012). Microfluidic synthesis of highly potent limit-size lipid nanoparticles for in vivo delivery of siRNA. Mol. Ther. Nucleic Acids.

[222] Kimura N., Maeki M., Sato Y., Ishida A., Tani H., Harashima H., Tokeshi M. (2020). Development of a microfluidic-based post-treatment process for size-controlled lipid nanoparticles and application to siRNA delivery. ACS Appl. Mater. Interfaces.

[223] Wei W., Sun J., Guo X.-Y., Chen X., Wang R., Qiu C., Zhang H.-T., Pang W.-H., Wang J.-C., Zhang Q. (2020). Microfluidic-Based Holonomic Constraints of siRNA in the Kernel of Lipid/Polymer Hybrid Nanoassemblies for Improving Stable and Safe In Vivo Delivery. ACS Appl. Mater. Interfaces.

[224] Henderson M.I., Eygeris Y., Jozic A., Herrera M., Sahay G. (2022). Leveraging biological buffers for efficient messenger RNA delivery via lipid nanoparticles. Mol. Pharm..

[225] Zhao P., Hou X., Yan J., Du S., Xue Y., Li W., Xiang G., Dong Y. (2020). Long-term storage of lipid-like nanoparticles for mRNA delivery. Bioact. Mater..

[226] Ball R.L., Bajaj P., Whitehead K.A. (2017). Achieving long-term stability of lipid nanoparticles: Examining the effect of pH, temperature, and lyophilization. Int. J. Nanomed..

[227] Zhang N.-N., Li X.-F., Deng Y.-Q., Zhao H., Huang Y.-J., Yang G., Huang W.-J., Gao P., Zhou C., Zhang R.-R. (2020). A thermostable mRNA vaccine against COVID-19. Cell.

[228] Zhang H., Leal J., Soto M.R., Smyth H.D., Ghosh D. (2020). Aerosolizable lipid nanoparticles for pulmonary delivery of mRNA through design of experiments. Pharmaceutics.

[229] Lamoot A., Lammens J., De Lombaerde E., Zhong Z., Gontsarik M., Chen Y., De Beer T.R., De Geest B.G. (2023). Successful batch and continuous lyophilization of mRNA LNP formulations depend on cryoprotectants and ionizable lipids. Biomater. Sci..

[230] Meulewaeter S., Nuytten G., Cheng M.H., De Smedt S.C., Cullis P.R., De Beer T., Lentacker I., Verbeke R. (2023). Continuous freeze-drying of messenger RNA lipid nanoparticles enables storage at higher temperatures. J. Control. Release.

[231] Muramatsu H., Lam K., Bajusz C., Laczkó D., Karikó K., Schreiner P., Martin A., Lutwyche P., Heyes J., Pardi N. (2022). Lyophilization provides long-term stability for a lipid nanoparticle-formulated, nucleoside-modified mRNA vaccine. Mol. Ther..

[232] Pardi M.L., Wu J., Kawasaki S., Saito H. (2022). Synthetic RNA-based post-transcriptional expression control methods and genetic circuits. Adv. Drug Deliv. Rev..

[233] Weissman D., Karikó K. (2015). mRNA: Fulfilling the promise of gene therapy. Mol. Ther..

[234] Munagala R., Aqil F., Jeyabalan J., Kandimalla R., Wallen M., Tyagi N., Wilcher S., Yan J., Schultz D.J., Spencer W. (2021). Exosome-mediated delivery of RNA and DNA for gene therapy. Cancer Lett..

[235] Ma C.-C., Wang Z.-L., Xu T., He Z.-Y., Wei Y.-Q. (2020). The approved gene therapy drugs worldwide: From 1998 to 2019. Biotechnol. Adv..

[236] Berraondo P., Martini P.G., Avila M.A., Fontanellas A. (2019). Messenger RNA therapy for rare genetic metabolic diseases. Gut.

